# Calcium Phosphate/Hyaluronic Acid Composite Hydrogels for Local Antiosteoporotic Drug Delivery

**DOI:** 10.3389/fbioe.2022.917765

**Published:** 2022-07-05

**Authors:** Alise Svarca, Andra Grava, Arita Dubnika, Anna Ramata-Stunda, Raimonds Narnickis, Kristine Aunina, Eleonora Rieksta, Martins Boroduskis, Inga Jurgelane, Janis Locs, Dagnija Loca

**Affiliations:** ^1^ Rudolfs Cimdins Riga Biomaterials Innovations and Development Centre of RTU, Institute of General Chemical Engineering, Faculty of Materials Science and Applied Chemistry, Riga Technical University, Riga, Latvia; ^2^ Baltic Biomaterials Centre of Excellence, Headquarters at Riga Technical University, Riga, Latvia; ^3^ Department of Microbiology and Biotechnology, Faculty of Biology, University of Latvia, Riga, Latvia

**Keywords:** calcium phosphate, osteoporosis, hyaluronic acid, hydrogels, strontium ranelate, composites

## Abstract

Despite the bone ability of self-regeneration, large bone defects require surgical intervention. Likewise, when it comes to osteoporotic bone fractures, new approaches should be considered a supportive mechanism for the surgery. In recent years, more and more attention has been attracted to advanced drug delivery systems for local osteoporosis treatment, combining appropriate biomaterials with antiosteoporotic drugs, allowing simultaneously to regenerate the bone and locally treat the osteoporosis. Within the current research, hyaluronic acid/strontium ranelate (HA/SrRan), HA/calcium phosphate nanoparticles (HA/CaP NPs), and HA/CaP NPs/SrRan hydrogels were prepared. The effect of CaP and SrRan presence in the composites on the swelling behavior, gel fraction, molecular structure, microstructure, and SrRan and Sr^2+^ release, as well as *in vitro* cell viability was evaluated. Obtained results revealed that the route of CaP nanoparticle incorporation into the HA matrix had a significant effect on the hydrogel gel fraction, rheological properties, swelling behavior, and microstructure. Nevertheless, it had a negligible effect on the release kinetics of SrRan and Sr^2+^. The highest cell (3T3) viability (>80%) was observed for HA hydrogels, with and without SrRan. Moreover, the positive effect of SrRan on 3T3 cells was also demonstrated, showing a significant increase (up to 50%) in cell viability if the used concentrations of SrRan were in the range of 0.05–0.2 μg/ml.

## 1 Introduction

Bone remodeling is strongly regulated by osteoblast and osteoclast crosstalk (Kim et al., 2020), and even small deviations in the natural balance between the bone-forming and bone-resorbing cells (when the last ones predominate) can result in osteoporosis (Yin et al., 2019). As osteosynthesis is compromised in osteoporotic bone, it can lead to prolonged fracture healing time (Kyllönen et al., 2015) and repeated fracture formation (Xie et al., 2019) that require surgical interventions (Ansari, 2019). Thus, it is important not only to find the right way to induce the bone regeneration processes but also to restore the balance between the activity of osteoclasts and osteoblasts. Strontium ranelate (SrRan) is an ideal candidate for that purpose, as it can simultaneously stimulate the activity of osteoblasts while suppressing the osteoclast differentiation (Pilmane et al., 2017). Nevertheless, the conventional drug delivery systems are sometimes insufficiently selective, or they are characterized with low oral bioavailability [e.g., 1–3% in the case of bisphosphonates (Aderibigbe et al., 2017; Papathanasiou et al., 2017; Farrell et al., 2018) and 19–27% in the case of SrRan (Briot and Roux, 2005)]. Due to this, high drug doses, which may induce toxic effects to other organs or tissues, are required to ensure sufficient drug concentrations in the defected bone site and also to reach the desired therapeutic effect (Hoare and Kohane, 2008; Chang et al., 2009).

In an effort to address these problems, in recent decades, scientists have focused on the controlled release drug delivery systems (CDDS) for local osteoporosis treatment. CDDS included such carriers as micro/nanoparticles (Stapleton et al., 2017; Farrell et al., 2018; Loca et al., 2018), polymeric and ceramic scaffolds (Asafo-Adjei et al., 2016), bone cement and composites (Chindamo et al., 2020), and hydrogels (Aderibigbe et al., 2017; Papathanasiou et al., 2017); all able to deliver unstable substances that can be easily degraded under the body conditions, as well as to provide guiding of the bioactive compounds directly to the desired site (Chang et al., 2009; Dreiss, 2020). These systems have been designed to overcome the challenges of the bone microenvironment, such as limited vascular perfusion near the bone surface (Farrell et al., 2018) and poor drug biodistribution in the bone (Newman and Benoit, 2016).

Hydrogels are three-dimensional polymer networks that can absorb large amounts of water or biological fluids, swell without dissolving, and are able to retain their three-dimensional structure (Kim et al., 2012; Fang et al., 2021). Hydrogels have easily modifiable properties; thus, it is possible to obtain desired release kinetics of a variety of active substances (Dreiss, 2020). Release kinetics of therapeutic agents can be controlled by modifying the mesh size of the polymer network and by ensuring links between the drug and the polymer chains. Additionally, the incorporation of another system into the hydrogel, such as micro and nanocapsules that contain active substances, has the ability to retard the active agent release (Li and Mooney, 2016).

Hyaluronic acid (HA) is the simplest glycosaminoglycan of high molecular weight, composed of natural polysaccharides, with repeated disaccharide (β-D-1,4-glucuronic acid-β-D-1,3-N-acetylglucosamine) units in the molecule (Ahmadian et al., 2019; Zhai et al., 2020). HA is biocompatible and completely biodegradable (Fang et al., 2021). HA within the human bone extracellular matrix (ECM) regulates cell adhesion, differentiation, and proliferation and controls cell–cell and cell–ECM interactions (Zhai et al., 2020). These attributes have attracted extended attention to HA and propelled its use in the synthesis of hydrogels and the development of drug delivery systems (Ahmadian et al., 2019). The main drawbacks of HA gels are their poor mechanical properties and rapid degradation *in vivo*. In order to overcome these shortcomings, HA polymer chains can be chemically crosslinked, thus forming a stable three-dimensional network (Kenne et al., 2013). 1,4-butanediol diglycidyl ether (BDDE) is a widely used crosslinking agent because of its biodegradability and negligible toxicity, compared to other ether-linking crosslinking agents, making BDDE safer for use in biomedicine. In the basic medium, stable ether bonds are formed in the reaction between the epoxide groups of the BDDE molecule and nucleophilic groups of the HA molecule (Zhang et al., 2018). The most likely reaction places are hydroxyl groups of HA as they are stronger nucleophiles than amide and carboxylic groups of HA (Yang et al., 2015). To form the cross-linkages, BDDE must react with HA on both ends, however, side reactions can occur and some part of the added BDDE can react with HA at one end and with water or hydroxide at the other end. This would lead to the formation of mono-linked BDDE or on the other hand, it could react only with water or hydroxide, thus forming hydrolyzed BDDE (Kenne et al., 2013).

The inorganic part of bone ECM is composed mainly of nanocrystalline hydroxyapatite (HAp); hence, synthetic calcium phosphates (CaP) are widely used in bone tissue engineering (Lin et al., 2020). Furthermore, CaP nanoparticles can be added during the preparation of hydrogels, providing not only a bone-mimetic microenvironment but also promoting bone regeneration and, at the same time, increasing the mechanical properties of obtained composite hydrogels (Sokolova et al., 2017; Choi et al., 2020).

Considering that strontium and calcium have similar physical and chemical properties (Kyllönen et al., 2015), the body is able to take up strontium in the tooth enamel and bone and replace the existing calcium (Sadegh, 2018), leading to the high interest for this divalent cation in the treatment of bone diseases (Pemmer et al., 2011; Marx et al., 2020). SrRan is a strontium (II) salt of ranelic acid accepted in several countries for the treatment of postmenopausal osteoporosis because of its ability to reduce vertebral and non-vertebral fracture risk (Marx et al., 2020).

Historically, Sr^2+^ in the structure of SrRan was considered to be an active component for bone regeneration. However, Yamaguchi and Neale Weitzmann (2012) provided *in vitro* evidence that only SrRan provides bioactivity by promoting osteoblast differentiation and inhibiting osteoclast formation but not its respective structures—sodium ranelate or strontium chloride. The efficacy of SrRan and strontium chloride in rats was also compared in the study by B. Pemmer, and the results verified that Sr uptake in the bone tissue of SrRan-treated animals was higher than that of Sr chloride-treated ones (Pemmer et al., 2011).

The mechanisms of SrRan action, underlying its effects on cells, have not yet been fully elucidated, but it is clear that SrRan possesses a unique dual mechanism of action. SrRan is available for systemic use and the oral dose of SrRan—2 g/day—seems to have the best effect in the treatment of osteoporosis (Kyllönen et al., 2015; Marx et al., 2020; Chen et al., 2021). Although low doses of SrRan stimulate bone formation, high doses of SrRan have an adverse effect on bone mineralization, potentially leading to decreased Ca absorption and possible changes in bone mineral properties. Prolonged use of SrRan can also cause a variety of side effects. The most common side effects are cardiovascular diseases, venous thromboembolism, myocardial infarction, gastrointestinal discomfort, hypersensitivity, diarrhea, nausea, dermatitis, and eczema (Kyllönen et al., 2015; Pilmane et al., 2017). Considering the various side effects of systemically administered SrRan, in recent years, efforts have been undertaken to develop new SrRan delivery systems that can deliver the active substance locally, in certain concentrations, to exactly the right place, thus not only reducing the risk of side effects but also accelerating the bone remodeling (Kyllönen et al., 2015).

Various SrRan carriers have been studied for this purpose, for e.g., SrRan-loaded poly (lactic-co-glycolic acid) (PLGA) microspheres with assembled silver and HAp nanoparticles for treating bone infections (Mao et al., 2018), polycaprolactone-laponite composite scaffolds as local SrRan delivery system (Nair et al., 2016), as well as polylactic acid microcapsules that released SrRan for more than 121 days (Loca et al., 2018). Even with the aforementioned efforts, the lack of research on HA hydrogels containing SrRan for local drug delivery is evident. Several studies can be found on strontium ion release from alginate (Place et al., 2011), carboxymethylcellulose (CMCA) (Nardone et al., 2012), photocrosslinked methacrylated alginate (PMA) (Zhao et al., 2021) and methoxy (polyethylene glycol)-polyester hydrogels (Peng et al., 2017), as well as injectable methylcellulose (MC) polymeric hydrogel (Chiang et al., 2021). It was shown that strontium ion release from the alginate hydrogels, in phosphate-buffered saline (PBS), had a rapid burst in the first 8 h and then gradually diminished in 5–10 days before settling to a constant rate (Place et al., 2011). From PMA hydrogels, a rapid burst was observed over the first day, and then the drug release was significantly slowed down until the seventh day (Zhao et al., 2021). Also, it was demonstrated that the release of strontium ions from CMCA hydrogels in an osteogenic medium enhanced the bone cell differentiation of PA2-E12 cell lines (Nardone et al., 2012), but in PMA hydrogels, osteogenic differentiation was observed for MC3T3-E1 cells (Zhao et al., 2021). In most of these studies, other strontium salts (SrCO_3_ and SrCl_2_) were used rather than ranelate (Chiang et al., 2021).

Bearing all the information in mind, the aim of our study was to develop and characterize novel hyaluronic acid hydrogels as local strontium ranelate delivery systems. HA hydrogels (HA H) and two types of hyaluronic acid/calcium phosphate (HA/CaP) hydrogels were prepared, where in one case CaP nanoparticles were added to the HA in the process of hydrogel synthesis (Mech_HA/CaP_H), but in the other case, CaP particles were synthesized directly in HA solution and the obtained composite material was used for the hydrogel (Synt_HA/CaP_H) preparation. All types of hydrogels were modified with SrRan, and composite properties such as swelling behavior, gel fraction, rheological properties, molecular structure, microstructure, drug release kinetics, and *in vitro* biocompatibility were evaluated.

## 2 Materials and Methods

### 2.1 Materials

Following reagents and materials were used in the current research: sodium hyaluronate (HA, cosmetic grade) with a molecular weight of 1.67 MDa (Contipro a.s., Dolní Dobrouč, Czech Republic); 1,4-butanediol diglycidyl ether (BDDE, ≥95.0%), phosphate-buffered saline (PBS), Dulbecco’s Modified Eagle Medium (DMEM), bovine calf serum (CS), fetal bovine serum, penicillin/streptomycin (P/S), dimethylsulfoxide (DMSO), neutral red (NR), and glacial acetic acid, (Sigma-Aldrich, St. Louis, MO, United States); strontium ranelate (SrRan) (Zhishang Industry Co., Ltd., Shandong province, China); sodium hydroxide (NaOH), sodium chloride (NaCl, 99.0–100.5%), and calcium oxide (CaO) (Merck KGaA, Darmstadt, Germany); orthophosphoric acid (H_3_PO_4_, 85%) (Chempur, Piekary Śląskie, Poland); and nitric acid (HNO_3_, 65%) (ChemLab, Zedelgem, Belgium).

### 2.2 Synthesis of Calcium Phosphate Nanoparticles

Calcium phosphate nanoparticles (CaP NPs) were synthesized using a slightly modified wet precipitation method, starting from calcium hydroxide and orthophosphoric acid, as described in the previous study (Salma et al., 2010). Briefly, to obtain 0.45 M Ca(OH)_2_ suspension, CaO was suspended in distilled water and homogenized at 500 rpm. Then, 2 M H_3_PO_4_ was added dropwise to the Ca(OH)_2_ suspension, at a slow addition rate (∼0.75 ml/min), and the obtained slurry was stirred at 500 rpm. The temperature of the synthesis was maintained constant at 45°C. The obtained precipitates were vacuum filtered, and the final content of calcium phosphate nanoparticles in the obtained suspension was 1.37 g/ml. The ending pH value of the suspension was 8.8.

### 2.3 Synthesis of Hyaluronic Acid/Calcium Phosphate Composite Material

CaP NP synthesis was performed *in situ* in HA solution using the wet precipitation method. Prior to HA/CaP synthesis, CaO powder was calcinated in a muffle furnace by first heating CaO for 3 h 18 min from 40°C to 1,100°C, followed by calcination at 1,100°C for 1 h and cooling for 3 h 18 min from 1,100°C to 40°C. Calcinated CaO was suspended in distilled water for 1 h at 300 rpm. The HA was slowly added to the calcium hydroxide suspension, providing heating of the mixture to 45°C. Better mixing of the resulting slurry was ensured by using an additional propeller-type impeller at 300 rpm for ∼30 min. Then, 0.2 M H_3_PO_4_ was added to the calcium hydroxide and HA suspension with a slow addition rate of 0.6 ml/min, under vigorous stirring, until reaching a Ca/P molar ratio of 1.67 and CaP/HA ratio of 60:40 wt%. The ending pH value of the suspension was 8–9. The heating of the mixture was stopped, and the resulting suspension was stirred for another 1 h at 300 rpm, frozen at −26°C, and lyophilized (BETA 2-8 LSCplus, Martin Christ Freeze Dryers, Osterode, Germany) for 72 h (pressure in the lyophilizer chamber was 1 mbar for primary drying stage and 0.0010 mbar for secondary drying stage).

### 2.4 Hydrogel Synthesis

Three different kinds of hydrogels were prepared, with initial compositions:

1. Hyaluronic acid hydrogels (HA H): 0.25 M NaOH (2020 μl) and BDDE (93 μl) were added to the HA (0.232 g) (HA 9 wt%, HA:BDDE 1:1).
2. Mechanical HA/CaP composite hydrogels (Mech_HA/CaP_H): BDDE (117 μl) and deionized water (611 μl) were added to the mixture of previously synthesized CaP nanoparticle suspension (2 g), NaOH (22.4 mg), and HA (0.258 g) (HA 9 wt%, HA:BDDE 1:1, CaP:HA 60 wt%:40 wt%).
3. *In situ* synthesized HA/CaP composite hydrogels (Synt_HA/CaP_H): 0.25 M NaOH (810 μl) and BDDE (37 μl) were added to the previously *in situ* synthesized HA/CaP composite material (0.2 g) (HA 9 wt%, HA:BDDE 1:1, CaP:HA 60 wt%:40 wt%).

The resulting reaction mixtures were homogenized for 2 h at room temperature, followed by hydrogel formation in stainless steel molds and by crosslinking of hydrogels at 45°C for 22 h. All obtained hydrogels were neutralized in 100 ml of 0.9% NaCl solution, at room temperature, for 48 h at 150 rpm; 0.9% NaCl solution was completely changed after 30 min, 1, 2, 3, and 24 h, and the pH of the obtained solution was measured. To determine the pH directly inside the hydrogel samples, pH-electrode (InLab® Micro, Mettler Toledo, CO, Ohaio, United States) was used and pH was recorded at different time points–0, 24, and 48 h. Neutralized hydrogels were then frozen at −26°C and lyophilized for 72 h (pressure in the lyophilizer chamber was 1 mbar for primary drying stage and 0.0010 mbar for secondary drying stage). Lyophilized samples were used for further studies.

### 2.5 Preparation of Strontium Ranelate Delivery Systems

For the preparation of SrRan containing hydrogels (HA H_SrRan, Mech_HA/CaP_H_SrRan, and Synt_HA/CaP_H_SrRan), see Section 2.4. Respectively, SrRan was added to the reaction mixture before the homogenization step, so that each sample contained 50 mg of SrRan. During the neutralization process, ∼20 ml of the changed 0.9% NaCl solution was frozen for further drug release studies.

### 2.6 Characterization of Prepared Samples

#### 2.6.1 Molecular Structure

Fourier transform infrared spectroscopy (FT-IR, Varian 800, Scimitar Series, Palo Alto, CA, United States) was used in the attenuated total reflectance mode (ATR, GladiATR™, PIKE Technologies, Fitchburg, WI, United States). By analyzing the molecular structure of lyophilized hydrogels, absorption bands of organic and inorganic phases were identified and possible phase interactions at the molecular level were determined. Absorbance was measured at 4 cm^−1^ resolution, in the wavenumber range between 400 and 4,000 cm^−1^ by co-adding 50 scans per sample.

#### 2.6.2 Phase Composition

To evaluate the phase composition of raw materials and prepared lyophilized hydrogel samples, X-ray diffraction analysis (XRD, PANalytical AERIS, Panalytical, Almelo, Netherlands) was used with Cu Kα radiation produced at 40 kV and 15 mA. XRD data were collected in a 10°–70° 2θ range, with a step size of 0.05° 2θ and time per step of 2.5 s. The PANalytical XPert Highscore 2.2 software (Panalytical, Almelo, Netherlands) as well as the International Centre for Diffraction Data PDF-2 (ICDD, Newtown Square, Pennsylvania, United States) database were used to analyze the obtained data. Before XRD analysis, CaP NPs suspension was lyophilized, and all samples were grounded into the fine powder by using a ball mill (Mini-Mill PULVERISETTE 23, FRITCH, Idar-Oberstein, Germany).

#### 2.6.3 Microstructure and Morphology

The morphology of lyophilized hydrogels was examined using scanning electron microscopy (SEM, Tescan Mira\LMU, Brno, Czech Republic), at an acceleration voltage of 5–7 kV. Each sample was attached to the sample holder with double-sided carbon tape. Prior to the examination, samples were sputtered with a thin layer (15–20 nm) of gold in sputter coater K550x (Quorum Technologies, Lewes, United Kingdom). Elemental analysis was performed with energy-dispersive X-ray spectroscopy (EDS) X-Max150 spectrometer (Oxford Instruments, Abingdon, United Kingdom) coupled with SEM. EDS spectra were taken at 15 kV using either single point or area analysis. Data were obtained and processed in Inca software (Oxford Instruments, Abingdon, United Kingdom).

High-resolution micro-computed tomography images were obtained using µCT 50 (Scanco Medical, Bassersdorf, Switzerland) instrument. The images were acquired at 70 kVp and 114 μ A, through a 0.5 mm thick aluminum filter. The voxel size of reconstructed 3D images was 7.4 µm × 7.4 µm × 7.4 µm. For all samples, the same size of volume of interest (VOI) was selected. Image processing was done by applying image processing language (IPL) developed by Scanco Medical. First, the obtained raw images were filtered with a Gaussian filter to reduce noise. Then, the polymer phase and air phase were segmented using image binarization by filtering voxels by their X-ray absorption. For the samples Mech_HA/CaP_H and Mech_HA/CaP_H_SrRan, filtering was applied twice to obtain one data set that included polymer phase and HAp agglomerate phase and one data set with only HAp agglomerate phase. Wall thickness and pore diameter histograms were obtained using the built-in “Bone Trab. Morphometry” script. The script applies distance transformation methods described in Hildebrand and Rüegsegger (1997). Images of the isolated pores were obtained by inverting the values of the segmented images and dividing the result into independent isolated objects, following the removal of the biggest object (the biggest object contains all the connected pores). The result was then filtered to remove very small pores (noise) and visualized within the original segmented image. Images for slightly isolated pores were obtained similarly as for the visualization of isolated pores. Segmented images were inverted; additionally, an erosion morphological image processing operation was applied to the biggest object by eroding five voxels off the surface of the connected pores. The pores that became isolated were divided into separate objects, and then five voxels were added back to the selected objects (morphological dilation). The results were then visualized within the original segmented image. Applying this image processing approach, the pores that were interconnected with the connections smaller than ≈74 μm were isolated and visualized.

#### 2.6.4 Rheological Characterization of Lyophilized Hydrogels

Discovery series HR20 rheometer (TA Instruments, DE, United States) was used for the characterization of rheological and mechanical properties of lyophilized hydrogels. Before the measurements, each lyophilized hydrogel sample was swollen in 20 ml of 0.01 M PBS for 2 h. An amplitude sweep test was performed in an oscillatory mode at 25°C using a 20 mm plate-plate geometry, shear strain varied from 0.01 to 1,000%, and a constant frequency of 1 Hz. A frequency sweep test was then performed in an oscillatory mode at 25°C using a 20 mm plate-plate geometry and frequency from 0.01 to 100 Hz at 0.2% strain. The working gap for amplitude and frequency sweep tests was adjusted for each sample separately. The silicon oil was spread around the sample prior to the measurements in order to avoid drying of the samples.

#### 2.6.5 Gel Fraction

The lyophilized hydrogel samples were weighed (W_o_) and placed in 200 ml of deionized water for 48 h to extract the uncross-linked polymer. The experiment was performed at room temperature at 100 rpm. After 48 h, the hydrogels were frozen at −26°C, lyophilized, and weighed (W_E_). Gel fraction (GF) was calculated according to Eq. 1:

$$GF\left(\%\right)=\frac{W_{E}}{W_{0}}\times 100,$$ (1)

where W_0_ is the initial weight of the dry hydrogel, g; W_E_ is the weight of the extracted dry hydrogel, g.

#### 2.6.6 Swelling Behavior

The solution uptake kinetics of the hydrogels was evaluated gravimetrically. The lyophilized hydrogel samples were weighed and placed in 20 ml of 0.01 M PBS at 37°C at 100 rpm. The samples were re-weighed at different time intervals: 30 min, 1, 2, 4, 6, 24, 48, 72 h, 7, 8, 9, 11, 17, 25, 32, and 45 days. The PBS for hydrogel samples was changed after 72 h, 11, 17, 25, 32, and 45 days. The degree of swelling was calculated using the following equation:

$$Q\left(\%\right)=\frac{W_{S}-W_{0}}{W_{0}}\times 100,$$ (2)

where W_S_ is the weight of the swollen sample, g; W_o_ is the initial weight of the dry sample, g.

#### 2.6.7 Strontium Ranelate Release Kinetics

The release kinetics of SrRan, from SrRan containing hydrogels, was determined during the neutralization process, and samples were collected when saline was changed (after 30 min, 1, 2, 3, 24, and 48 h). Standard and sample solutions were measured against 0.9% NaCl in a two-beam ultraviolet-visible light spectrophotometer (UV/VIS, Evolution 300, Thermo Scientific, Waltham, MA, United States) at *λ* = 318 nm and expressed as cumulative SrRan release from samples. SrRan content in the dissolution medium was determined using a five-point calibration curve over the concentration range from 0.8 to 80 μg/ml. Standard sample preparation included SrRan dissolution in 0.9% NaCl, for 10 min, in an ultrasonic bath with subsequent stirring on a magnetic stirrer for 40 min at 600 rpm.

#### 2.6.8 Strontium Release Kinetics

To evaluate the release kinetics of Sr^2+^ from the hydrogels during the neutralization process (samples were collected when saline was changed after 30 min, 1, 2, 3, 24, and 48 h), inductively coupled plasma mass spectrometry (ICP-MS) was used. Lyophilized hydrogel samples were dissolved in deionized water and high-purity nitric acid solution. To complete the reaction, samples were kept for 20 min at room temperature. Then, samples were transferred to a Mars 6 microwave oven (CEM Corporation, Matthews, NC, United States) where the temperature was increased up to 150°C, within 30 min and held for 30 min at 150°C. The solution, cooled to room temperature, was filtered through a filter with a pore size of 12–15 μm (Filtres Fioroni, Ingré, France), quantitatively transferred to volumetric flasks, and diluted to 50 ml with deionized water. An Agilent 7700x ICP-MS instrument with Mass Hunter Workstation software for ICP-MS (version B.01.03, Tokyo, Japan) was used for the analysis of elements.

Strontium content in the hydrogel sample was calculated using the following equation:

$$m_{Sr^{2+}}\left(g\right)=\frac{2\times M_{Sr^{2+}}}{M_{SrRan}}\times m_{SrRan},$$ (3)

where $m_{Sr^{2+}}$ is the weight of Sr^2+^ in the hydrogel sample, g; $M_{Sr^{2+}}$ is molecular weight of Sr^2+^, g/mol; M_SrRan_ is molecular weight of SrRan, g/mol; m_SrRan_ is the weight of the SrRan in the hydrogel sample.

#### 2.6.9 *In Vitro* Biocompatibility of Hydrogels

Samples were sterilized with water vapor sterilization at 105°C by using the electronic tabletop autoclave ELARA 11 (Tuttnauer, Breda, Netherlands).

Cytotoxicity of three types of hydrogels, after the neutralization with and without SrRan (Section 2.4) were tested on NIH 3T3 and L929 mouse fibroblasts and human osteoblasts MG-63.

For extract test, 5,000 cells per well were seeded in a 96-well plate in 200 μl cell medium which consisted of 89% Dulbecco’s Modified Eagle Medium (DMEM), supplemented with 10% (v/v) calf serum for 3T3 cell line, 10% fetal bovine serum for L929, and MG-63 cells and 1% penicillin/streptomycin (P/S). PBS (pH 7.4) was added to the wells around the perimeter to prevent the solution on the plate from drying out. Prior to the experiment, the plates with the cells were incubated overnight at 37°C, with 5% CO_2_ (New Brunswick™ S41i CO_2_ Incubator Shaker, Eppendorf, Hamburg, Germany).

Each hydrogel sample was immersed in a fresh 2 ml of cell medium. After 2, 4, 6, 24, and 48 h in the case of NIH 3T3 tests or after 2 and 24 h in the case of L929 and MG-63, all the solution was collected from the samples and replaced with fresh 2 ml cell medium. The collected solution was used as undiluted extract and diluted with fresh medium (extract to cell medium 1:10 and 1:100) and immediately put onto the preincubated cells (200 μl for each well). Untreated cells were used as a positive control, while a 5% dimethylsulfoxide (DMSO) solution in the medium was applied to cells as a negative control. There were six replicates for each treatment.

To assess the cytotoxicity of hydrogel extracts and their dilutions, the neutral red (NR) test was used (Stolarczyk et al., 2021). After 24 h of incubation for each time point, extracts and their dilutions in the cell medium were removed and cells were washed with 200 μl PBS solution, which was followed by the addition of 150 μl NR (25 μg ml^−1^) in 5% serum-containing cultivation media to each well and incubation at 37°C, with 5% CO_2_. After 3 h, NR media was removed and 1% glacial acetic acid/50% ethanol solution was added to extract the dye accumulated in viable cells. After 20 min of incubation at room temperature, absorption at 540 nm was measured using an Infinite M Nano microplate reader (Tecan, Männedorf, Switzerland).

#### 2.6.10 Hemocompatibility of Hydrogels

Hemocompatibility studies were performed in accordance with the approval of the Committee of Research Ethics of the Institute of Cardiology and Regenerative Medicine, University of Latvia. A hemolysis test was performed to assess the hemocompatibility of hydrogels and 24 h hydrogel extracts. Blood from healthy donors was collected in vacutainers containing ethylenediaminetetraacetic acid (EDTA) (S-Monovette®, Sarstedt, Nümbrecht, Germany). Blood was diluted with 0.9% sodium chloride solution (4:5 ratio by volume). Hydrogel samples were washed three times with PBS (pH 7.4) and added to 15 ml tubes containing fresh 9.8 ml of PBS and tubes incubated at 37°C, with 5% CO_2_ for 30 min. In the case of hydrogel extracts, 9.8 ml of 20% extracts were used. Extracts were prepared as described in Section 2.6.9, and 0.2 ml of diluted blood was added to each tube and incubated at 37°C, with 5% CO_2_ for 1 and 8 h. PBS was used as a negative control and deionized water as a positive control. After incubation, tubes were centrifuged at 2,000 rpm for 5 min, the supernatants were collected, and the absorbance was measured at a wavelength of 545 nm using an Infinite M Nano microplate reader.

#### 2.6.11 *In Vitro* Cytotoxicity of SrRan

To evaluate the effect of SrRan on cell proliferation after 24 and 48 h, SrRan solution in full cell medium was tested at various concentrations on 3T3 mouse fibroblast cells. Cells were seeded according to the same procedure as for the hydrogel extracts (Section 2.6.9) and left overnight in the incubator. After 24 h, cell medium was removed, and cells were treated with 2; 1.5; 1.0; 0.5; 0.2; 0.1; and 0.05 μg/ml SrRan solutions in full cell medium, 150 μl per well. SrRan concentrations in the cell medium were selected based on the drug release data. A 5% DMSO solution in cell medium was applied as a negative control, while untreated cells were used as a positive control. Each treatment had six replicates. Cell metabolic activity was measured with an NR assay.

### 2.7 Statistical Evaluation

All results were expressed as the mean value ± standard deviation (SD) of at least three independent samples. The significance of the results was evaluated using an unpaired Student’s *t*-test with the significance level set at *p* < 0.05. The *in vitro* biocompatibility data were analyzed using Microsoft Excel software (Microsoft 153 Corporation, Redmond, WA, United States). One-way ANOVA analysis was used to test for differences among groups with *p* < 0.05 considered as a significant difference. To note the significant differences in results, Tukey HSD tests were performed**.**

## 3 Results and Discussion

### 3.1 Characterization of the Prepared Hydrogels

**FIGURE 1:**
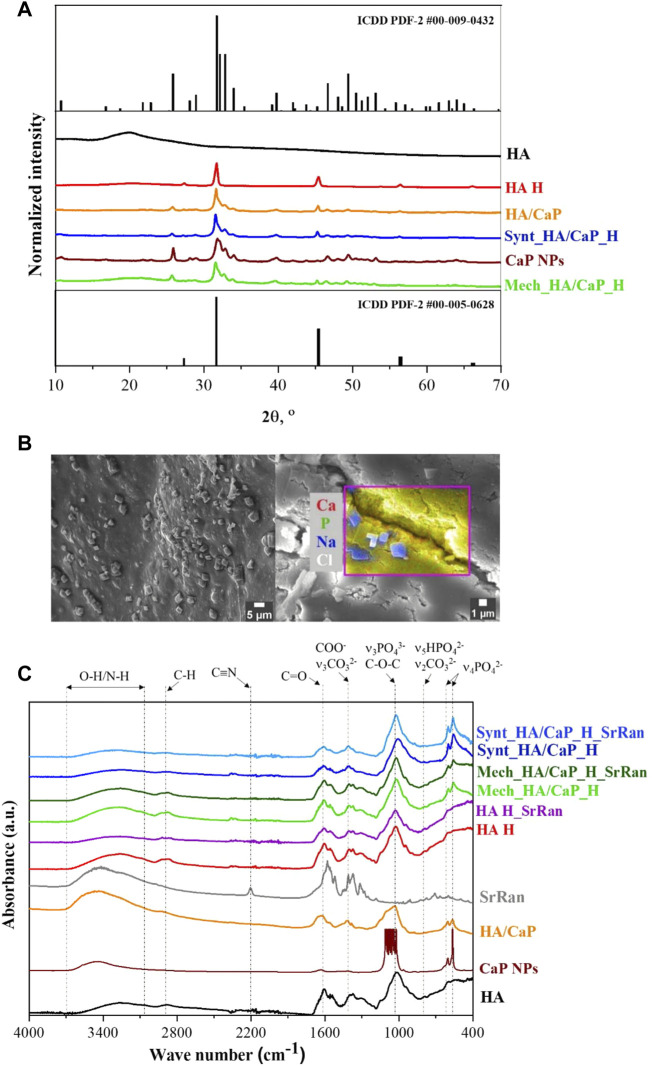
Phase composition and molecular structure of prepared composites. **(A)** XRD patterns of raw materials and prepared lyophilized hydrogels; **(B)** SEM micrograph and EDS area analysis revealing NaCl crystals on the surface of Mech_HA/CaP_H sample; **(C)** FT-IR spectrum.

Crystallinity and phase composition of raw materials and prepared samples were assessed by XRD (Figure 1A).

HAp phase was confirmed for both lyophilized CaP NPs and *in situ* synthesized HA/CaP composite material (ICDD PDF-2 #00-009-0432). Comparing diffraction patterns of CaP NPs and HA/CaP composite material, it was observed that the addition of HA increased the crystallinity of HAp. The same conclusion was made by Chanthick et al., who determined that HA increases calcium oxalate crystallinity (Chanthick and Thongboonkerd, 2020). Also, Chen et al. (2012) have found that HA plays a crucial role in the CaP crystallization process, preventing CaP nanoparticles from agglomeration. Additionally, diffractions patterns of lyophilized hydrogels showed characteristic reflections (27.4°, 31.7°, 45.4°, 53.7°, 56.4°, 57.3°, and 66.2° 2θ) corresponding to halite NaCl (ICDD PDF-2 #00-005-628). The presence of NaCl was also confirmed by EDS analysis. NaCl crystals (∼2 μm in size) were detected on the surface of all types of hydrogel samples (Figure 1B), formed after the hydrogel neutralization process in a physiological solution. To remove excess NaCl, hydrogel samples could be rinsed with deionized water after the neutralization process. Furthermore, HAp phase was not affected during HA/CaP composite hydrogel preparation process. Nevertheless, the presence of NaCl produced sharper maxima at 31.7° 2θ in the diffractogram of Mech_HA/CaP_H and Synt_HA/CaP_H samples. Analyzing HA diffraction pattern, broad reflection centered at 20° 2θ was observed, indicating its non-crystalline structure (Hamad et al., 2017), however, in the diffraction patterns of HA containing hydrogels, the broad characteristic reflection of HA was not well detectable. Most probably, it was suppressed by the pronounced reflections of NaCl and HAp structures.

FT-IR analysis was performed in order to identify the characteristic functional groups of the prepared hydrogels, as well as to evaluate the possible interactions between the components. The presence of CaP and apatite-like structure in HA/CaP hydrogels was confirmed by absorption bands at 960, 600^,^ and 555 cm^−1^ (Figure 1C).

These bands correspond to the absorption of PO_4_^3−^ groups, indicating the presence of CaP in samples (Sokolova et al., 2017). Also, the FT-IR spectra of pure raw materials, *in situ* synthesized HA/CaP composite material and CaP NPs dried at 100°C, were analyzed, and characteristic absorption bands of PO_4_^3−^ group (at 960, 600, and 555 cm^−1^) were observed.

The wide band in the 3,100–3,600 cm^−1^ region could be attributed to the valence vibrations of the hydroxyl group O-H and N-H of HA (Sokolova et al., 2017). In the FT-IR spectra of CaP, the relevant wide band 3,100–3,600 cm^−1^ is attributed to the valence vibrations of both the adsorbed water and the phosphate O-H groups (Andrade Neto et al., 2016).

In the FT-IR spectrum of HA and HA hydrogels, the bands at around 1,600 and 1,400 cm^−1^ are characteristic of asymmetric (C=O) and symmetric (C-O) valence vibrations of hyaluronate carboxyl groups (Sokolova et al., 2017). The medium intensity bands were observed around 2,920 cm^−1^, which appeared due to the C-H valence vibrations. Bands around 1,560 and 1,380 cm^−1^ appeared due to amide II and amide III vibrations, respectively. The line around 1,020 cm^−1^, which overlaps with the PO_4_^3−^ characteristic absorption band, corresponds to the C-O-C (O-bridge) group within the HA molecule (Alkrad et al., 2003; Sokolova et al., 2017).

SrRan characteristic absorption band at 2,200 cm^−1^ in the prepared active substance delivery systems was not clearly distinct. This could be attributed to the fact that FT-IR analysis was performed after the neutralization process of hydrogels, so some part of the active substance (105 ± 19% from HA H_SrRan, 48 ± 12% from Synt_HA/CaP_H_SrRan, and 44 ± 8% from Mech_HA/CaP_H_SrRan) was already released, and drug concentration in the sample was not sufficient to distinguish SrRan characteristic absorption band against the background of HA.

Previously, it has been reported that PO_4_^3−^ has a high affinity to amine groups but Ca^2+^ to carboxyl, hydroxyl, phosphate, and sulfate groups; therefore, these interactions are widely used to obtain composite materials with desired properties (Farbod et al., 2014). The carboxyl groups in HA could bind Ca^2+^, and it has been stated that such interactions affect the bonding strength of the carboxyl group double bond, which in FT-IR spectra could be characterized by intensity change and absorption band shifts of C=O and COO− groups. In the research by R. H. Ellerbrock et al., the interaction between Ca^2+^ and carboxyl groups of polygalacturonic acid (PGA) molecule was studied by FTIR, and it was revealed that the addition of Ca^2+^ to PGA causes the formation of COO− groups, resulting in an increased intensity of COO− absorption and decreased intensity of COOH band. It was also observed that C=O and COO− absorption bands were shifted toward the lower wavenumbers (Ellerbrock and Gerke, 2021). However, as sodium hyaluronate is used within this study, the COO- group is already formed in the HA molecule and the intensity change of C=O and COO− groups upon the addition of Ca^2+^ are difficult to determine. Also, characteristic bond frequencies of the components were not altered, thus FT-IR results did not confirm any new chemical bonds or molecular interactions between the components of hydrogels.

**FIGURE 2:**
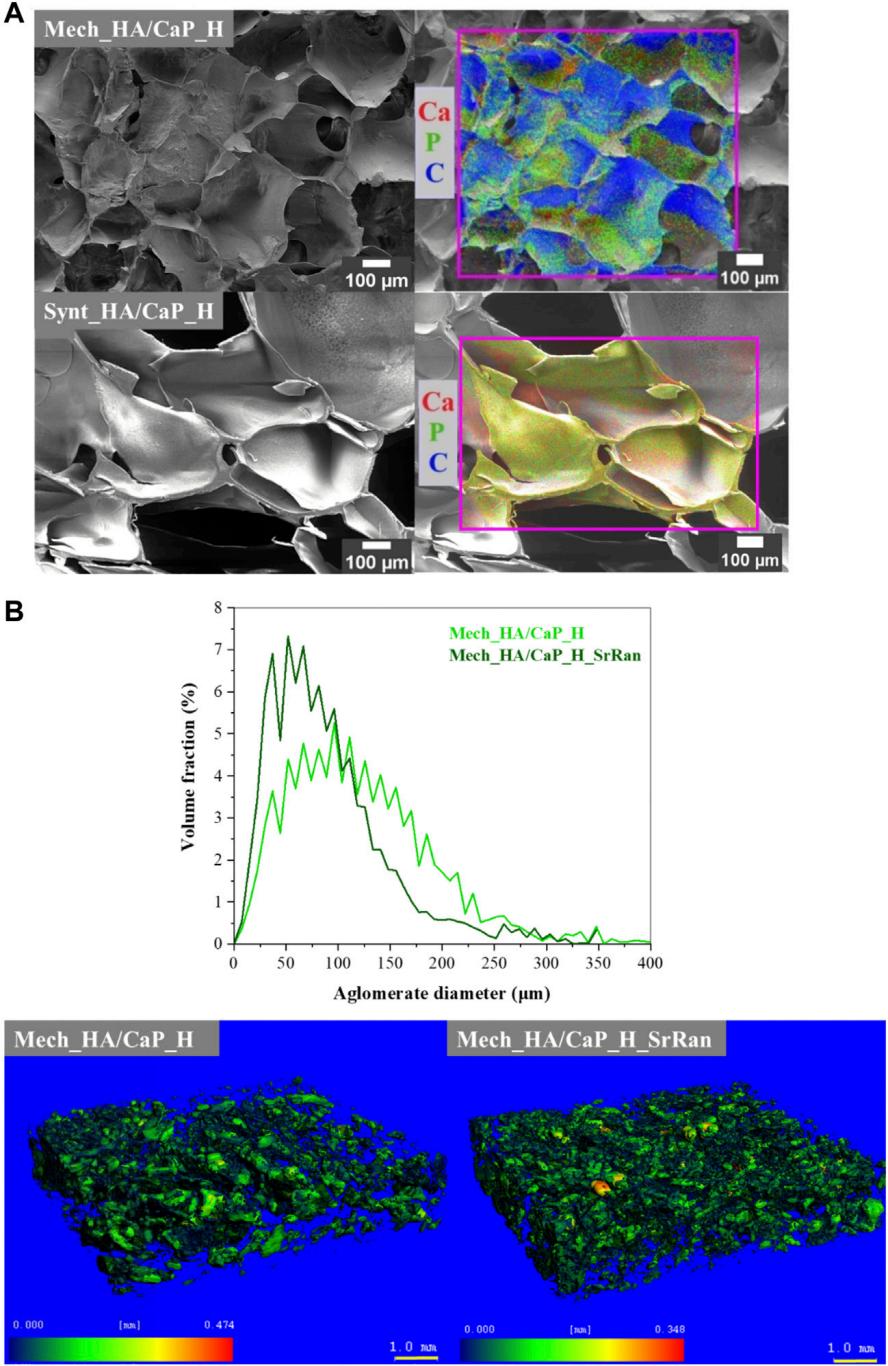
CaP distribution within the HA matrix. **(A)** SEM-EDS microphotographs; **(B)** volume fraction as a function of agglomerate diameter of CaP agglomerates in the lyophilized hydrogels and heatmap of CaP agglomerates’ size distribution in the hydrogel matrix.

To identify the elements of prepared HA-containing composites and to evaluate the CaP distribution within the composite matrix, EDS analysis was performed. The obtained results (Figure 2A) confirmed the presence of Ca, P, and C in both Mech_HA/CaP_H and Synt_HA/CaP_H samples. Even distribution of Ca, P, and C was found for Synt_HA/CaP_H sample, thus indicating homogeneous CaP particle distribution within the composite.

To evaluate the distribution of CaP in the hydrogel matrix, μ-CT and image processing were also applied. The distribution of CaP in the HA matrix could not be revealed in the cases of Synt_HA/CaP_H and Synt_HA/CaP_H_SrRan by the μ-CT used, as CaP/HA composite material presumably consists of CaP nanoparticles that are uniformly distributed throughout the HA matrix. It has been reported previously that HA can be used as a template for CaP crystal growth, controlling their size and morphology like in the native bone biomineralization process. In the study by Z. Chen et al., CaP nanoparticles were precipitated in an HA matrix by an aqueous ammonia diffusion co-precipitation method, and it was revealed that HA inhibits agglomeration of CaP crystals. Inhibition was initiated by first entrapping Ca^2+^ into the HA matrix due to the complexing interactions and then surrounding newly formed CaP crystals into loops of HA (Chen et al., 2012). A similar process could be observed during *in situ* HA/CaP composite material synthesis, thus homogeneously distributing non-agglomerated CaP nanoparticles within the HA matrix.

During Mech_HA/CaP_H and Mech_HA/CaP_H_SrRan synthesis, already prepared CaP NP suspension was mechanically mixed with HA. Inefficient mixing leads to the CaP NP agglomerate formation 40–250 μm in size (Figure 2B).

As the raw materials of Mech_HA/CaP hydrogels are mechanically mixed, slight differences in CaP agglomerate size distribution can be observed among both samples.

**FIGURE 3:**
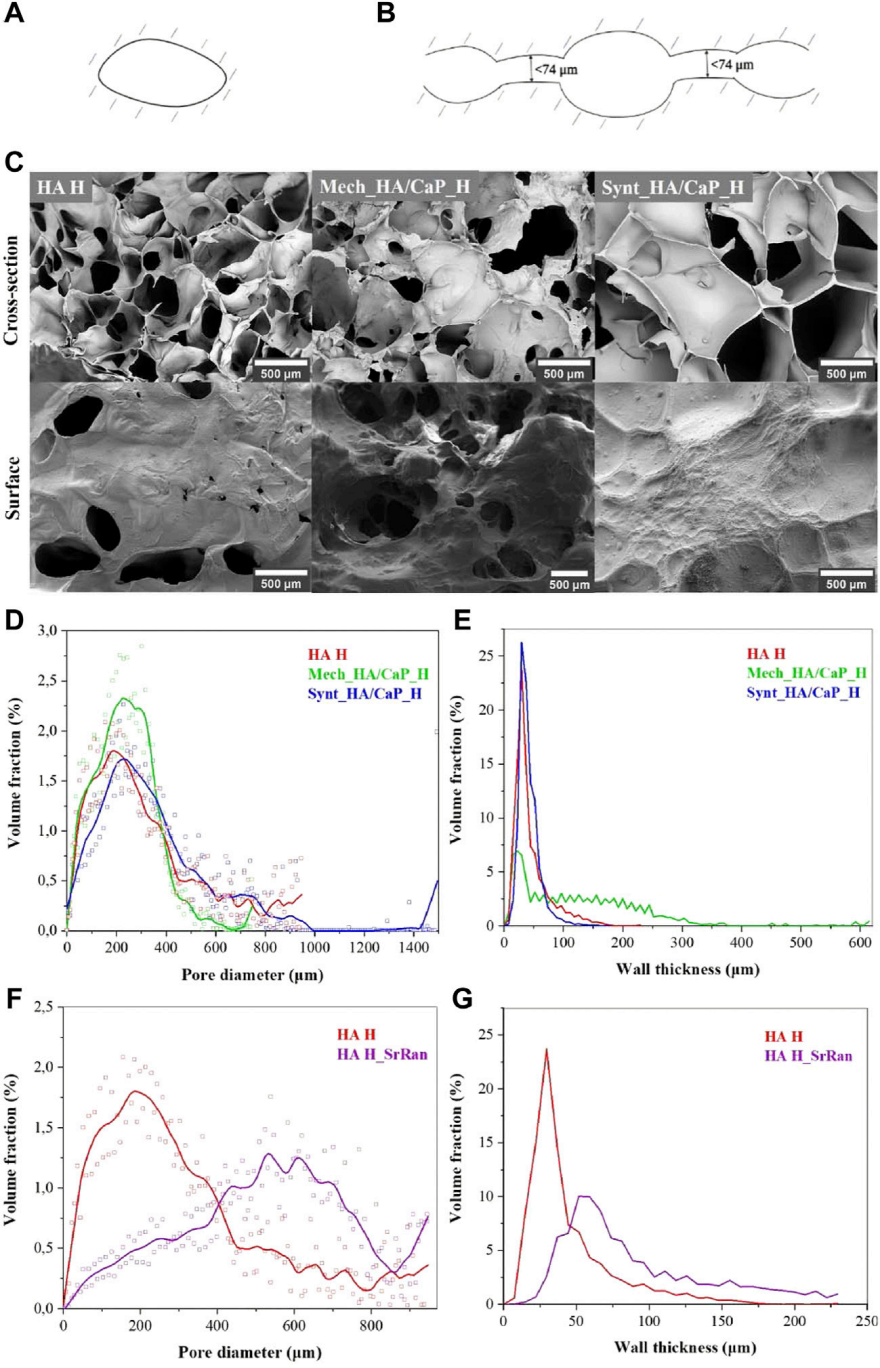
Characterization of HA-containing hydrogel microstructure. **(A)** Illustration of closed pores unclonable by bone cells; **(B)** illustration of slightly connected pores unclonable by bone cells; **(C)** SEM microphotographs of HA-containing hydrogels; **(D)** pore volume fraction as a function of pore size in the lyophilized hydrogels; **(E)** volume fraction of pore walls as a function of wall thickness in the lyophilized hydrogels; **(F)** pore volume fraction as a function of pore size in the lyophilized HA and HA_SrRan hydrogel; **(G)** volume fraction of pore walls as a function of wall thickness in the lyophilized HA and HA_SrRan hydrogel.

Cell infiltration, migration, proliferation, and differentiation within the scaffold depend on the pore size and their interconnectivity (Abbasi et al., 2020). It has been reported that pores larger than 300 μm are desirable to enhance the ingrowth of bone cells and blood vessels within the material, thus accelerating bone regeneration (Karageorgiou and Kaplan, 2005; Zhen et al., 2010). However, smaller pores (<100 μm) cause the formation of the fibrous tissue instead of bone tissue (Abbasi et al., 2020). Therefore, SEM, μ-CT, and image processing were applied to characterize the surface and cross-section of lyophilized hydrogel samples. Pore size distribution in the composite, pore wall thickness, as well as pore interconnectivity (revealing closed and slightly connected pores, where cells cannot migrate through and colonize the whole volume of the implant), were analyzed. As the size of osteoblasts can range from 20 to 50 μm (Abbasi et al., 2020), the pores with junctions of less than 74 μm (slightly connected pores) were considered isolated pores (Figures 3A,B).

Obtained SEM results indicated that the HA-containing hydrogels have a macroporous structure with interconnected pores (Figure 3C).

The formation of macropores in the structure of the hydrogels can be explained by the formation of ice crystals during the freezing of the samples before lyophilization. Moreover, the pore size and their distribution highly depend on the cooling rate during freezing. At this stage, the free water and possibly the part of the chemically bound water turn into the ice crystals, which, as they grow, form matrix-free regions and push the polymer chains closer together. During the lyophilization, sublimation of ice crystals occurs, leading to the formation of macropores (Grenier et al., 2019).

The results showed that for all samples, most of the pores are in the range of 100–400 μm and 86% of pores, in the total pore volume, were greater than 100 μm in the case of HA H, 85% in the case of Mech_HA/CaP_H, and 90% in the case of Synt_HA/CaP_H (Figure 3D).

The pore wall thickness is in the range of 10–60 μm for HA H (84% of the total volume of the pore wall thickness) and Synt_HA/CaP (94% of the total volume of the pore wall thickness) (Figure 3E). However, for Mech_HA/CaP hydrogels, pore walls were thicker, and 89% of pore walls were 10–250 μm thick because of the CaP aggregates located in the pore walls.

For SrRan-containing composites, macroporosity and interconnection of pores were also observed. The pore size distribution in the lyophilized samples did not differ significantly from the corresponding composites without active substance in the case of Mech_HA/CaP_H and Synt_HA/CaP_H (Supplementary Figure S1). However, differences in the pore size distribution and pore wall thickness between HA H and HA H_SrRan samples were observed (Figures 3F, G).

Since SrRan is already released from HA H_SrRan hydrogels during the neutralization process (Section 3.3), the active substance is unlikely to affect the structure formation of the hydrogels during the freezing, thus it is not clear why such a difference occurred.

**FIGURE 4:**
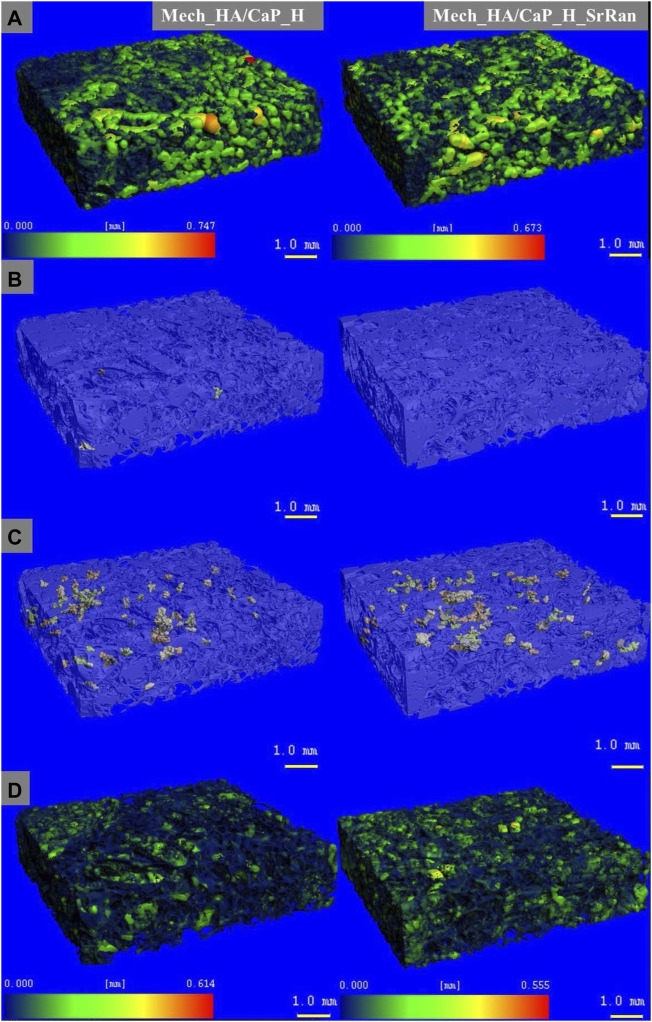
Microstructure visualization of Mech_HA/CaP hydrogels. **(A)** Heatmap of pore size distribution; **(B)** closed pores; **(C)** slightly connected pores; **(D)** heatmap of pore wall thickness.

Heatmap of the pore size distribution and pore wall thickness, as well as closed pores and slightly connected pores of Mech_HA/CaP hydrogels, are presented in Figure 4.

It was found that the total closed pore volume of the Mech_HA/CaP_H sample was 0.02%, and no closed porosity was found in Mech_HA/CaP_H_SrRan sample. For both samples, slightly connected porosity was detected and was 0.7 and 1.1% of total pore volume for Mech_HA/CaP_H and Mech_HA/CaP_H_SrRan samples, respectively.

Microstructure visualization of HA and Synt_HA/CaP hydrogels are presented in Supplementary Figures S2 and S3. HA H and HA H_SrRan samples showed closed porosity of 0.02 and 0.01% of total pore volume, but slightly connected porosity reached 2.8 and 0.2%, respectively. However, Synt_HA/CaP_H and Synt_HA/CaP_H_SrRan samples showed significantly higher closed porosity, reaching 4.3 and 1.6%, whereas slightly connected porosity was 17.03 and 0.6% of the total pore volume.

Obtained results revealed that the developed HA-containing lyophilized hydrogels could ensure the migration of bone cells and the formation of new bone tissue within its matrix, as the pore size is in the range of 100–400 μm. The Synt_HA/CaP_H samples showed the highest total closed porosity of 21.3%, which could complicate the cell migration and colonization within the whole implant volume.

### 3.2 Influence of CaP Addition on Hyaluronic Acid Hydrogel Properties

As the hyaluronic acid gels have low mechanical properties and are known to rapidly degrade *in vivo*, covalently crosslinked three-dimensional polymer networks were obtained using BDDE as a crosslinking agent. To mimic the native bone composition and increase bioactivity, CaP NPs were added to the HA during hydrogel preparation. Obtained results revealed that the addition of CaP, as well as the route of nanoparticle introduction into the HA matrix, highly influenced the gel fraction of composites. Furthermore, gel fraction strongly affected the pH changes and swelling of the hydrogels during their neutralization process, as well as the swelling behavior of the lyophilized samples. However, statistically significant differences in composite properties were not observed between samples with and without SrRan modifications.

**FIGURE 5:**
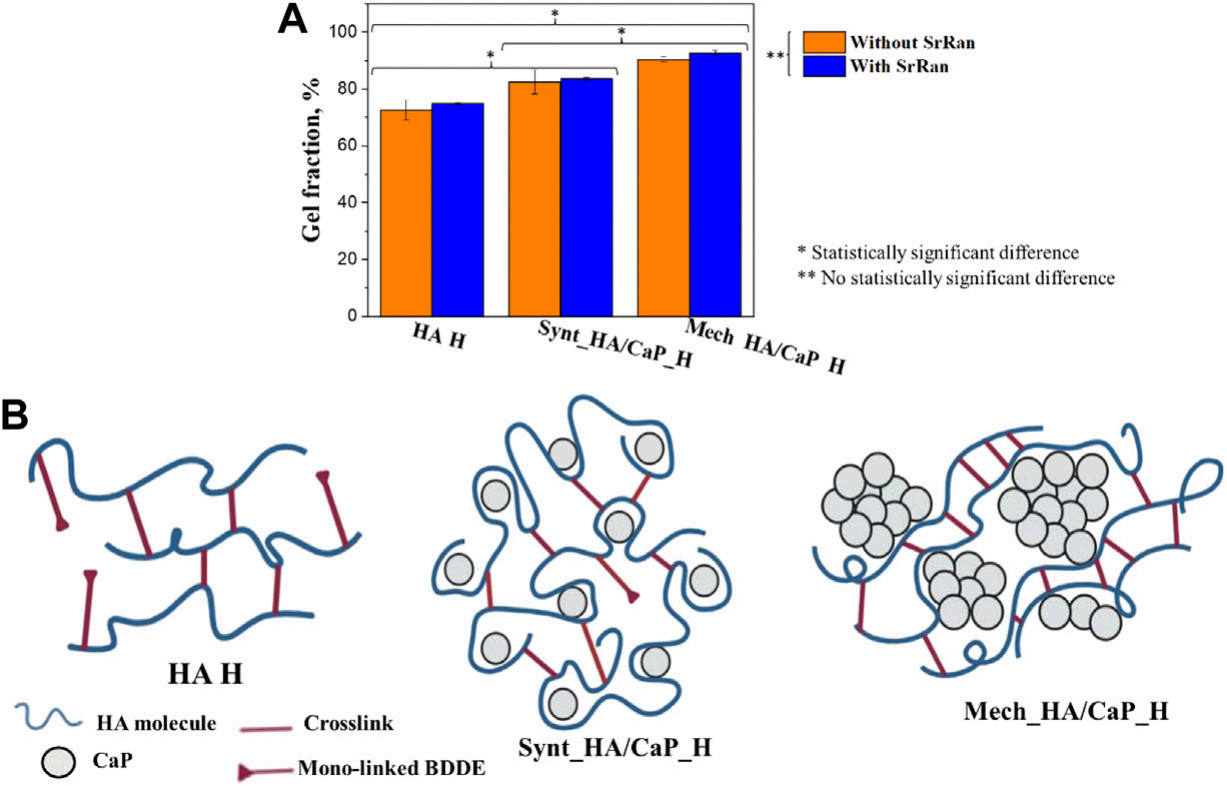
Influence of CaP addition on the crosslinking degree of hyaluronic acid-containing hydrogels: **(A)** gel fraction of HA-containing composites; **(B)** schematic illustration of possible crosslink formation in HA-containing hydrogels.

It was found that the Mech_HA/CaP_H composites have the highest crosslinking degree (90 ± 1%) and it significantly differed from the gel fraction of Synt_HA/CaP_H (82 ± 4%) and HA H (72 ± 3%) composites (Figure 5A).

As it was mentioned before, to form the crosslinks, BDDE must react with HA at both ends. We suppose that the CaP addition to HA enhanced the gel fraction of hydrogels by moving the HA molecules closer to each other, thus creating more favorable conditions for crosslinking reactions. Without CaP additive, mono-linked or hydrolyzed BDDE could be formed as HA molecules are not close enough to form the crosslinks (Figure 5B).

On the other hand, gel fraction could also be influenced by the approach used for CaP particle incorporation into the HA matrix. The homogeneously distributed CaP particles would provide a sufficiently close distance between HA molecules, thus facilitating the formation of crosslinks during the preparation of Synt_HA/CaP_H hydrogels (Figure 5B). Furthermore, during Mech_HA/CaP_H synthesis, it is possible that CaP agglomerates push the HA molecules even closer to each other (Figure 5B), thus forming a more crosslinked structure and resulting in an increased gel fraction value.

**FIGURE 6:**
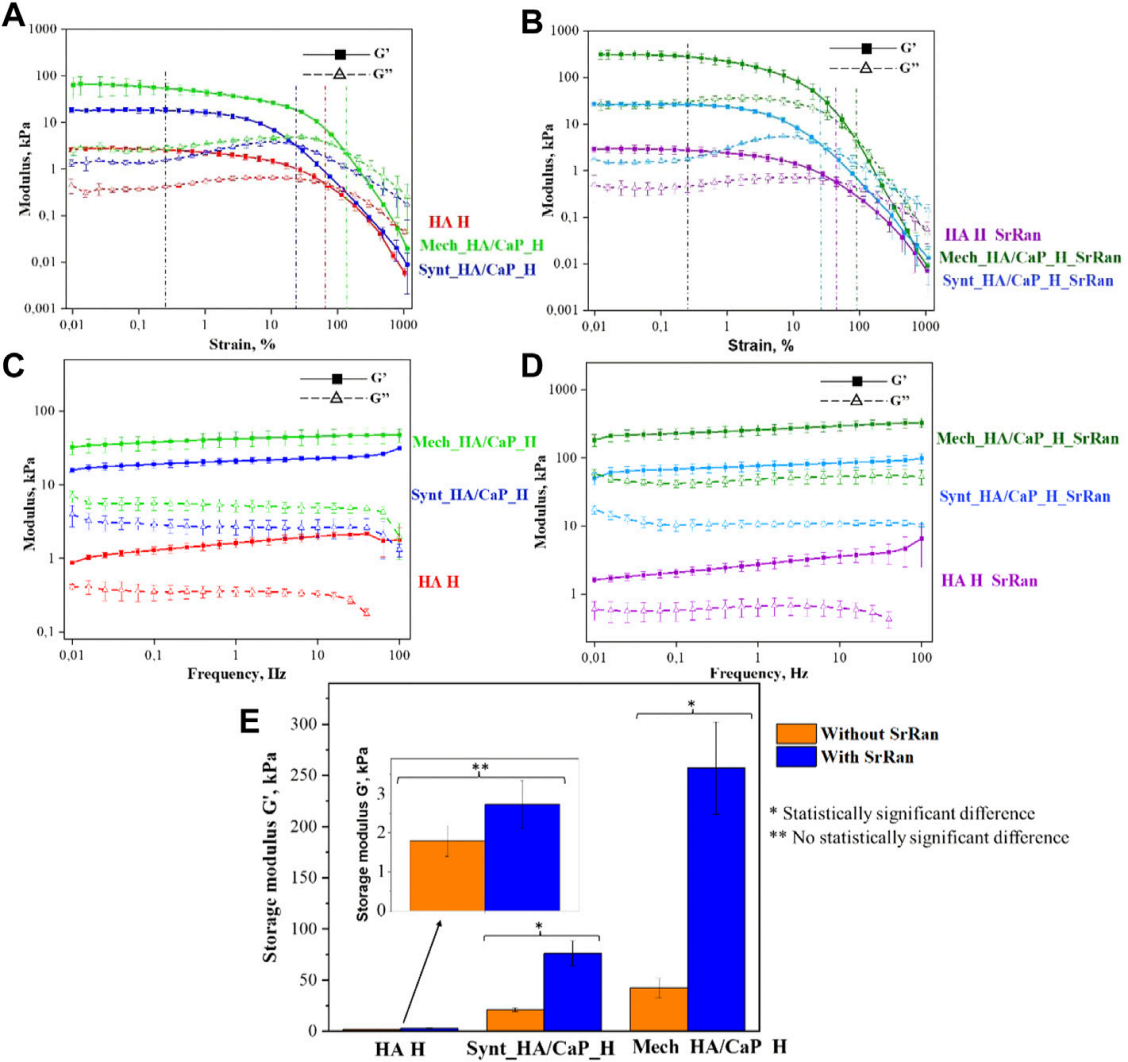
Oscillatory rheology. **(A)** Amplitude sweep test of HA-containing hydrogels without SrRan obtained at 1 Hz frequency; **(B)** amplitude sweep test of HA-containing hydrogels with SrRan obtained at 1 Hz frequency; **(C)** frequency sweep test of HA-containing hydrogels without SrRan obtained at 0.2% strain; **(D)** frequency sweep test of HA-containing hydrogels with SrRan obtained at 0.2% strain; **(E)** extracted mechanical stiffness of HA-containing hydrogels from frequency sweeps at 1 Hz and at 0.2% strain.

The results obtained from rheological measurements were used to evaluate the mechanical properties of lyophilized hydrogels impregnated with PBS, thus mimicking the structure of hydrogels as it would transpire during implantation. All types of chemically crosslinked hydrogels showed typical gel-like behavior, as the storage modulus was significantly higher than the loss modulus (G′ > G″) (Figures 6A–D).

The amplitude sweep test revealed a well-defined linear viscoelastic region (LVER) in which the moduli are independent of increasing the strain. After LVER, as the strain was increased, the storage moduli (G′)–loss moduli (G″) crossover point was reached, indicating the strain at which the material starts to behave like a fluid (Stojkov et al., 2021). The chemically cross-linked hydrogels exhibited LVER until a strain ε ≈ 0.25%, marked with a black vertical line (Figures 6A, B). In the amplitude sweeps, the differences in the mechanical behavior of different kinds of hydrogels were observed by the significantly different G′/G″ ratio, which was the highest for Mech_HA/CaP_H (indicating more solid-like behavior). However, the G′/G″ ratio was around 1.5 and 3 times lower in the case of Synt_HA/CaP_H and HA H samples, respectively. Moreover, the addition of SrRan showed a higher loss modulus in the case of Mech_HA/CaP_H and Synt_HA/CaP_H, indicating less viscous flow behavior, while the G′/G″ ratio was not affected by the addition of SrRan. The average crossover point was slightly different for all types of hydrogels, marked with colored lines (Figures 6A, B). In the case of Synt_HA/CaP_H and Synt_HA/CaP_H_SrRan, G′–G″ crossover point was at strain ε ≈ 20%, whereas in the case of HA H and HA H_SrRan, the values reached ε ≈ 72% and ε ≈ 45%, respectively. At the same time, Mech_HA/CaP_H and Mech_HA/CaP_SrRan started to behave like a fluid at ε ≈ 135% and ε ≈ 85%, respectively. As it was previously reported, during *in situ* HA/CaP composite material synthesis, HA could be used as a template, surrounding newly formed CaP crystals into loops of HA (Chen et al., 2012). For this reason, the elasticity of HA could be reduced by limiting the mobility of polymer molecules. This would explain why Synt_HA/CaP_H hydrogels lost their solid-like structure at lower strain.

After identifying LVER in the amplitude sweep experiment, the frequency sweep test was carried out at 0.2% shear strain. The BDDE crosslinked hydrogels showed behavior similar to the previously reported chemically crosslinked HA hydrogels (Gu et al., 2017; Salma-Ancane et al., 2022), where G′ is constant across all the frequency ranges (Figures 6C, D), thus demonstrating solid-like and stable inner structure. However, HA H, HA H_SrRan, Mech_HA/CaP_H, and Synt_HA/CaP_H exhibited a decrease in G″ modulus at frequencies higher than 60 Hz, possibly due to the breakage of the samples or due to the limitations of the system measurements for G″, which don’t have the same range as the G′ (Figures 6C, D).

Additionally, the storage modulus was extracted from the frequency sweeps [an approach used previously for both physically and chemically crosslinked hydrogels (Wychowaniec et al., 2020; Salma-Ancane et al., 2022)], thus evaluating the mechanical stiffness of all lyophilized hydrogels (Figure 6E). As expected from the observed gel fraction results, Mech_HA/CaP_H exhibited a significantly higher storage modulus value (42 ± 9 kPa) than Synt_HA/CaP_H (21 ± 2 kPa) and HA H (2 ± 0.4 kPa). Although the presence of SrRan did not affect the gel fraction of hydrogels, the addition of the active substance significantly increased the stiffness of Mech_HA/CaP_H_SrRan (257 ± 45 kPa) and Synt_HA/CaP_H_SrRan (76 ± 12 kPa). The addition of SrRan did not affect the stiffness of HA H due to the fact that oscillatory rheology was done after the neutralization process, during which all the drug was already released from HA_H_SrRan samples.

**FIGURE 7:**
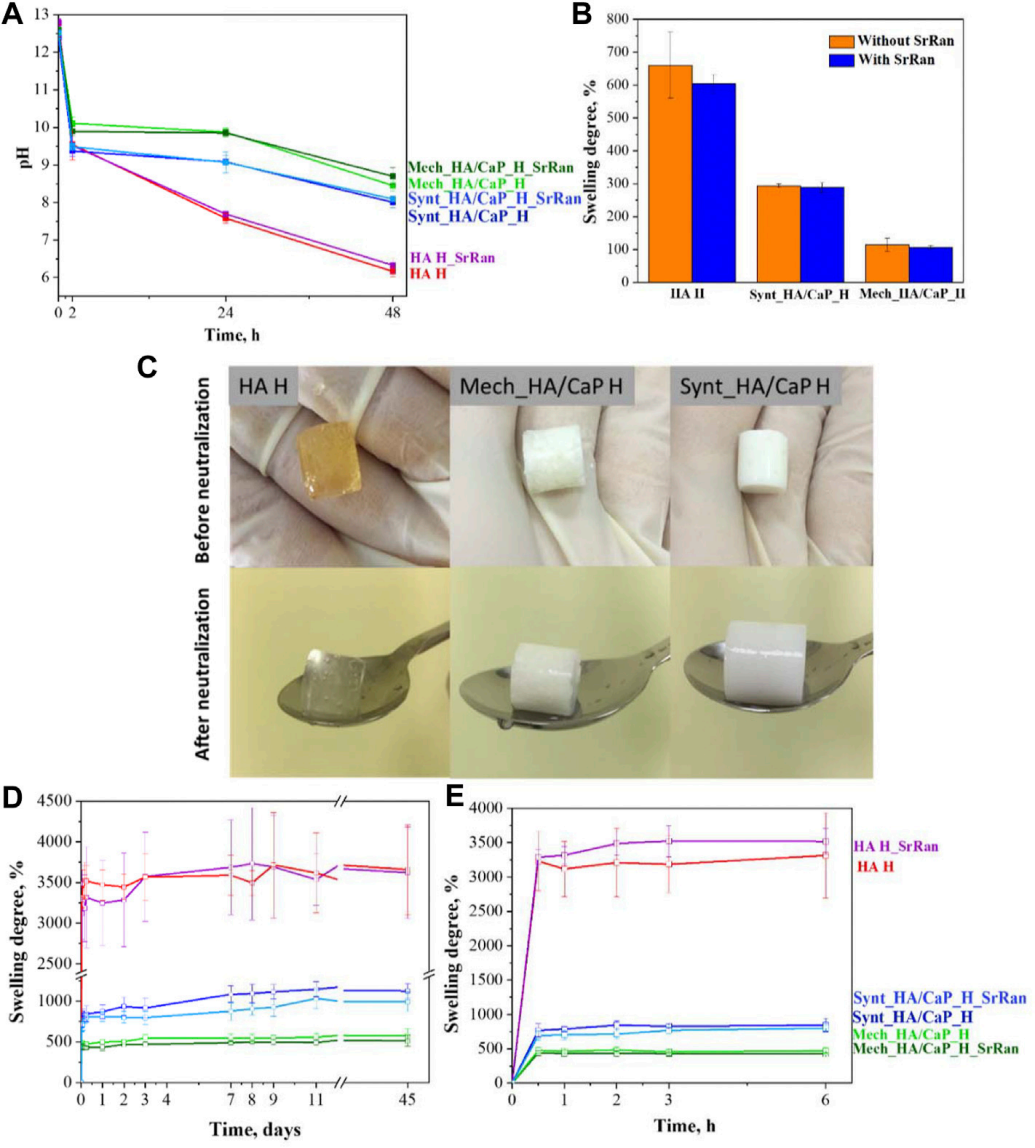
Neutralization process and swelling degree of hyaluronic acid-containing hydrogels. **(A)** pH change of HA-containing hydrogels during neutralization; **(B)** swelling ratio of hydrogels during neutralization; **(C)** HA-containing hydrogels before and after neutralization; **(D)** swelling behavior of HA-containing lyophilized hydrogels for 45 days; **(E)** swelling behavior of HA-containing lyophilized hydrogels in the first 6 h.

Since the reaction of HA and BDDE took place in a basic medium (pH ∼ 10), it was necessary to neutralize the hydrogels before *in vitro* investigations; 0.9% NaCl solution (pH ∼ 5.8) was used for the hydrogel neutralization, and the pH of the solution was measured after 2, 24, and 48 h. To evaluate the neutralization kinetics in bulk, pH inside the hydrogels was measured at the beginning of the neutralization process (0 h), followed by measurements after 24 and 48 h (Figure 7A).

Obtained results indicated that hydrogels were strongly basic before the neutralization (pH ∼ 12.5 at 0 h) and it was more difficult to neutralize CaP-containing hydrogels as the neutralization solution still showed a basic environment after 48 h (pH 8.45 ± 0.15 for Mech_HA/CaP_H and pH 8.01 ± 0.14 for Synt_HA/CaP_H), compared to HA hydrogels which became weakly acidic (pH 6.16 ± 0.14). There were no statistically significant differences found between pH values measured in the solutions and pH values measured inside the samples, thus both methods can be applied for the pH determination during the neutralization process.

Within the neutralization process, the hydrogels absorbed saline and swelled (Figure 7B). This is the characteristic ability of hydrogels to absorb water and biological fluids up to thousands of times their dry weight (Li and Mooney, 2016). During swelling, the distance between HA chains increases, so sodium hydroxide and unreacted residues can be more effectively released from the hydrogel samples. The obtained results (Figure 7B) correlated with the results of gel fraction (Figure 5A)—the higher the degree of hydrogel crosslinking, the tighter the polymer network, the smaller the swelling ratio and the slower the release of basic environment and unreacted residues from the samples.

Hydrogel samples were also evaluated visually before and after their neutralization (Figure 7C). It was observed that hyaluronic acid hydrogels (HA H) changed their color from yellow (before neutralization) to colorless (after neutralization). The yellow color after the crosslinking could indicate the presence of unreacted residues that were leached out during the neutralization process. It has been previously reported that sodium hyaluronate degrades (separates monomers from the polymer chain) at high pH values as well as at elevated temperatures. It is also known that high molecular weight HA chains degrade more slowly than low molecular weight chains (Lowry and Beavers, 1994; Lapčík et al., 1998). As the crosslinking reaction took place in a basic medium and at 45°C, the unreacted residues were degraded by staining the sample, but in the neutralization process, these unreacted residues were eluted and hydrogels became colorless.

Solution uptake kinetics was evaluated for lyophilized samples, and it was observed that the addition of CaP decreased the swelling ratio of samples by about three and six times in the case of Synt_HA/CaP_H and Mech_HA/CaP_H, respectively (Figure 7D).

Results revealed that the swelling trend of HA-containing lyophilized hydrogels observed during the first 48 h within the neutralization process (Figure 7B), continued for at least 45 days.

It was also observed that the weight of hydrogels increased rapidly in the first hour, after which a swelling equilibrium was reached (Figure 7E). All analyzed samples were stable during the observation period of 45 days, confirming their stability and integrity in the PBS environment, however, the enzyme hyaluronidase is involved in the breakdown of HA in the body (Žádníková et al., 2022), so the stability of hydrogels should also be tested *in vivo*.

### 3.3 Release Kinetics of Sr^2+^ and Strontium Ranelate

**FIGURE 8:**
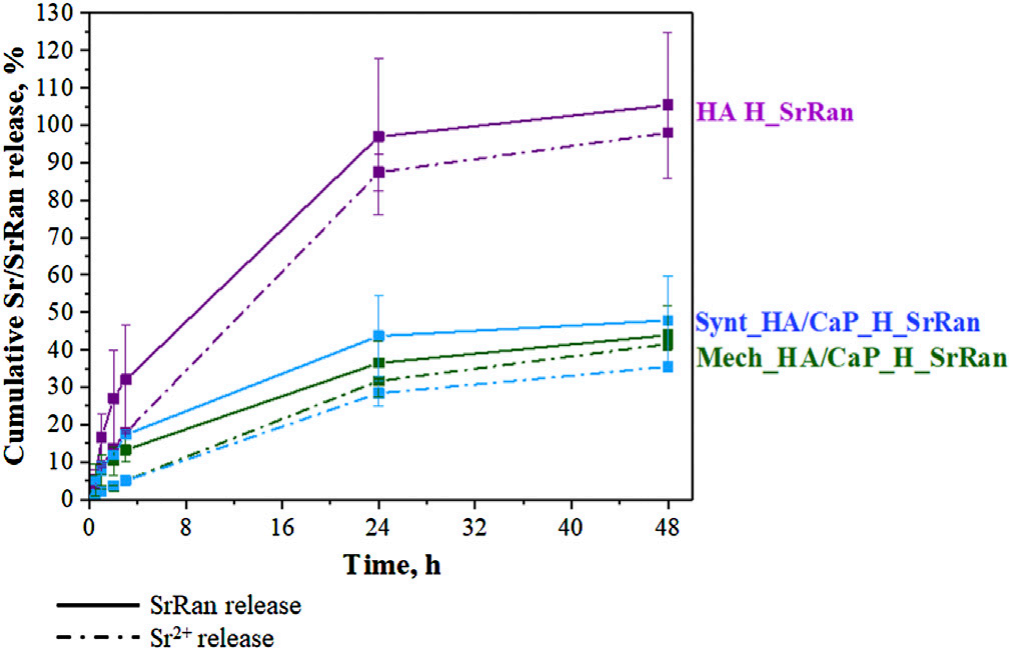
Cumulative SrRan and Sr^2+^ release during the neutralization process of hydrogels.

Each prepared sample initially contained an equal amount of SrRan. SrRan release kinetics were studied for 48 h during the hydrogel neutralization process. As the experiments were performed in 0.9% NaCl solution, the stability of SrRan molecule was also evaluated by measuring the Sr^2+^ release with ICP-MS and compared to the SrRan release measured by UV-VIS (Figure 8).

No statistically significant differences between the cumulative release of SrRan and Sr^2+^ were found, suggesting that both methods UV/VIS and ICP-MS can be applied for the determination of SrRan release from prepared hydrogels.

Hyaluronic acid hydrogels (HA H_SrRan) exhibited considerable SrRan burst release within the first 24 h (Figure 8), probably due to the initial release of drug molecules on or near the surface of the sample (Wei et al., 2018), and already after 48 h, the entire drug was released. The burst release was also influenced by the morphology of hydrogels such as pore size and distribution, as well as the degree of crosslinking. In general, the burst release of the active substance will be lower if the hydrogel is more crosslinked (smaller size of meshes) and has a smaller pore size (Huang and Brazel, 2001). The SrRan release studies of HA H_SrRan were in good agreement with gel fraction, neutralization, and swelling degree results, which revealed that hydrogels with the least crosslinked polymer network can absorb up to six times more solution than Ca-containing hydrogels.

The addition of CaP to the hydrogels retarded the SrRan release by ∼60%, and it was found that after 48 h, 48 ± 12% and 44 ± 8% of SrRan were released from Synt_HA/CaP and Mech_HA/CaP hydrogels, respectively. Obtained results revealed that although the route of CaP nanoparticle incorporation into the HA matrix had a significant effect on the hydrogel gel fraction and swelling behavior, it had a negligible effect on the release kinetics of the active substance.

### 3.4 *In Vitro* Evaluation of Lyophilized Hydrogels

Cytotoxicity of hydrogel extracts was evaluated with 3 cell lines (NIH 3T3, L929 mouse fibroblasts, and human osteoblasts MG-63) that are the most commonly used for testing the safety of osteoregenerative materials.

**FIGURE 9:**
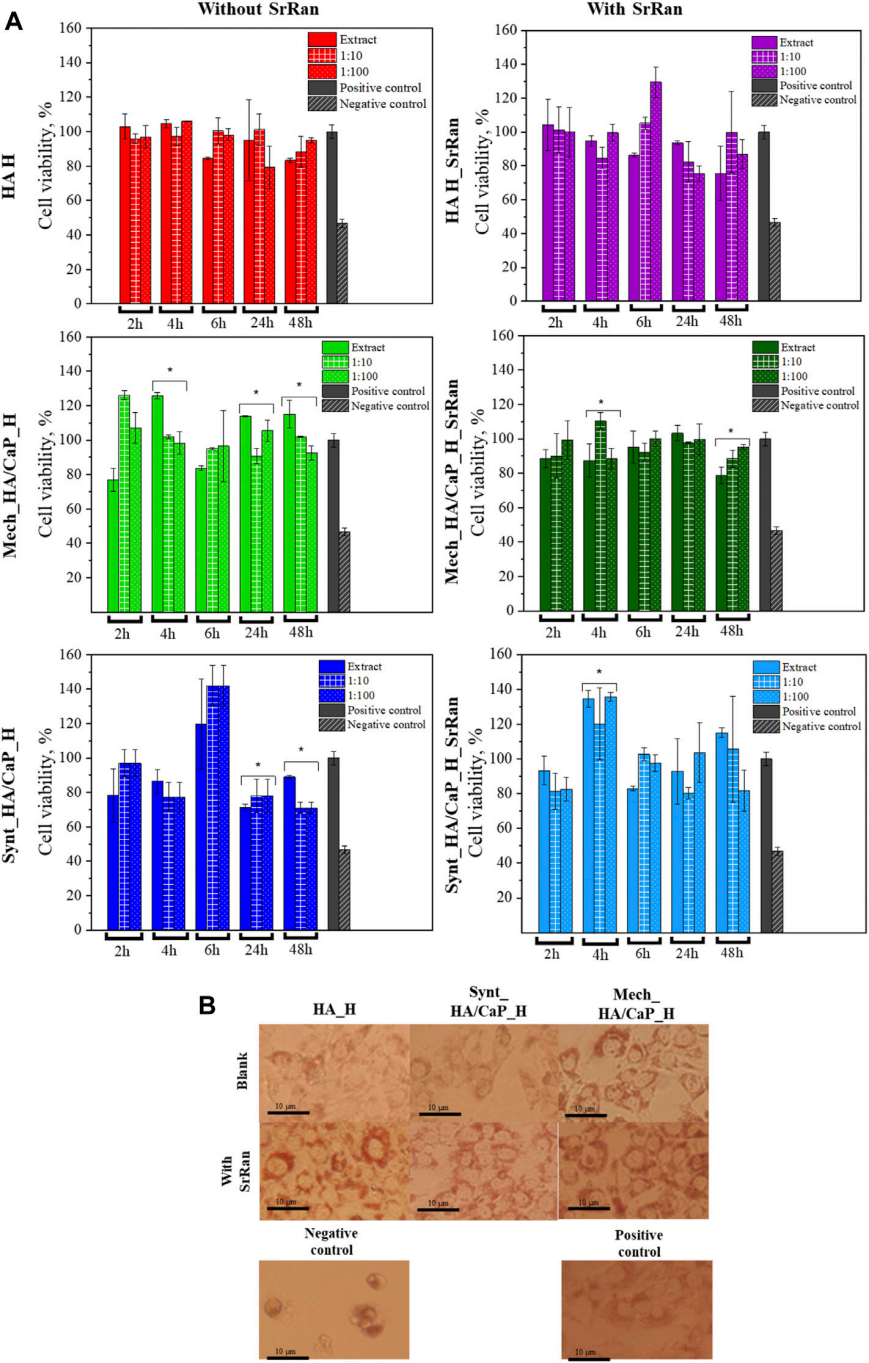
*In vitro* evaluation of lyophilized hydrogels. **(A)** Cytotoxicity of obtained hydrogels after neutralization (*annotate the statistical difference at a *p* < 0.05); **(B)** morphology of 3T3 cells treated with hydrogel extracts after 24-h incubation.

3T3 cell response to the obtained, neutralized, and lyophilized hydrogels is shown in Figure 9A.

To compare the obtained data, negative and positive controls were used. Positive control (cells treated with full cell medium, with no modifications) is presented as gray bars, and negative control (cells treated with 5% DMSO in cell medium) is presented as bars with a slash. Cell morphology after 24 h is presented in Figure 9B. When compared to the positive control, there was no significant alteration in the cell morphology of 3T3 cells for all hydrogel extracts. A closer look at the cell morphology at each time point and dilution can be found in the supporting data section (Supplementary Figures S4–S7).

None of the tested extracts and their dilutions showed cytotoxicity. In all experiments, cell viability was higher than 70%. According to ISO 10993-5:2009, a cytotoxicity effect is considered if the cell viability is reduced by more than 30%. Dilutions did not show significant differences if compared to extracts in most of the experiments, however, statistically significant differences are indicated (*p < 0.05). There was no specific trend found in the differences between the extract and their dilutions, indicating that the dilution can both lower and elevate the cell viability if compared to the pure extract.

The highest cell viability was observed for HA hydrogels with and without SrRan (more than 80% cell viability for all time points), while the lowest cell viability was found for hydrogels prepared from synthesized HA/CaP composite. As HA is known to promote the cell viability due to its interaction with membrane receptors CD44 and CD168, external cell membrane interaction with HA through these receptors modulates the cell proliferation and transformation of such growth factors as β-TGF and CTGF (Mariggiò et al., 2009; Mandal et al., 2019). At the same time, decreased cell viability for CaP-containing hydrogels could be attributed to the pH of the hydrogels, as moderately alkaline pH (Section 3.2) could lead to biofilm formation, which in turn increases cell death (Kruse et al., 2017).

**FIGURE 10:**
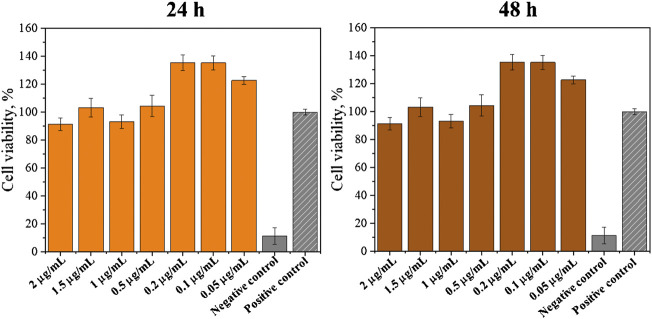
SrRan effect on 3T3 cell proliferation (*annotate the statistical difference at a *p* < 0.05).

Additionally, experiments for the determination of the SrRan effect on 3T3 cells were performed. Obtained results are presented in Figure 10.

It was observed that SrRan increased the cell proliferation when concentrations of 0.05–0.2 μg/ml were used. This explained why the hydrogels with SrRan showed higher cell viability when compared with hydrogels without SrRan addition. The same effect of a strontium-containing drug on fibroblast growth was observed by Fernandes et al. (2019) who concluded that strontium citrate increases the human gingival fibroblast activity. Er et al. (2008) also observed that SrRan does have a favorable effect on fibroblast cells at low concentrations. SrRan was associated with inducing fibroblast growth factor (FGF) signaling pathways, which are independent of calcium-sensing receptor (CaSR) (Caverzasio and Thouverey, 2011). Also, extracellular Ca^2+^ ions could enhance fibroblast proliferation (Mcneil et al., 1998), hence increased Ca^2+^ and Sr^2+^ ion concentrations from the hydrogels have a significant effect on cell reproduction.

**FIGURE 11:**
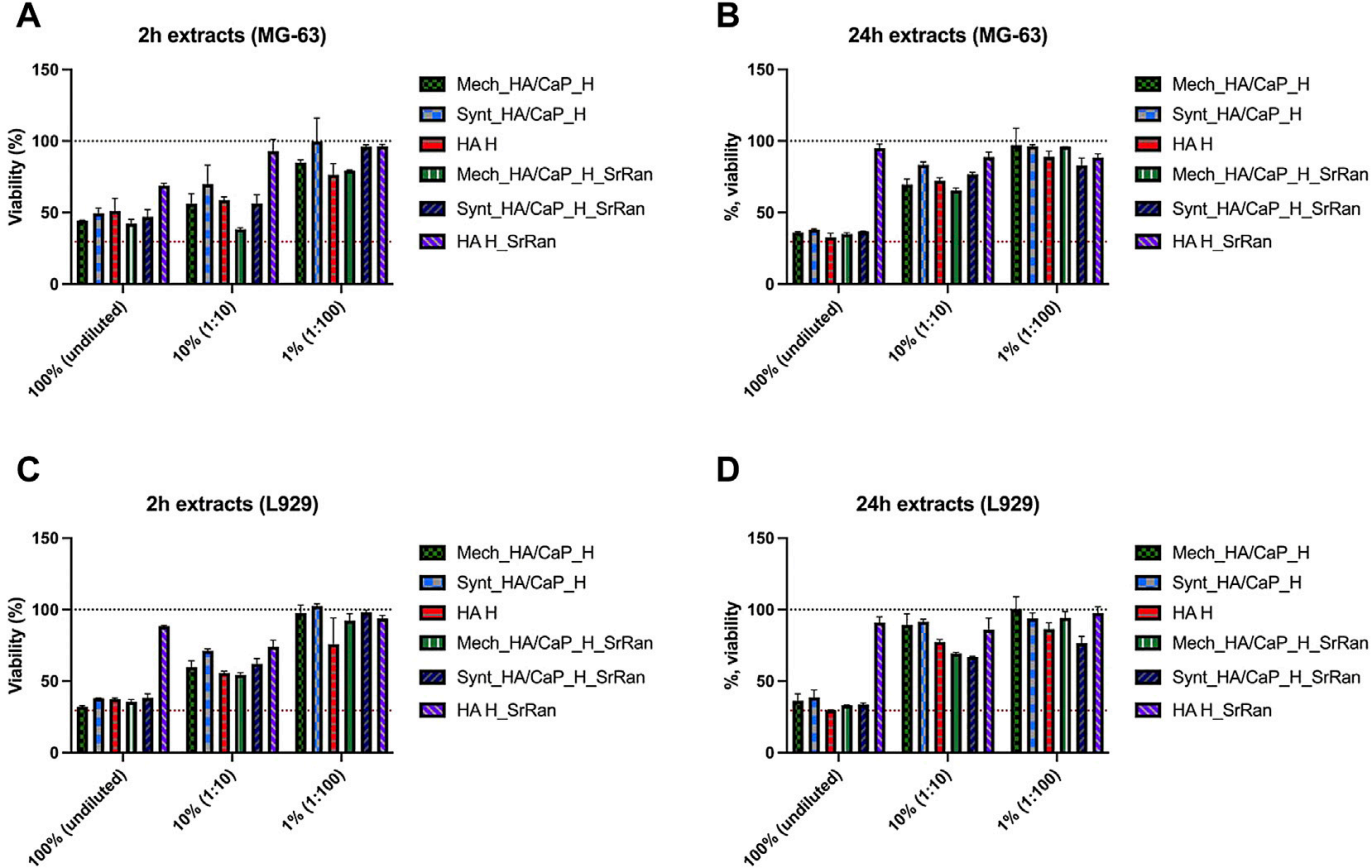
Cytotoxicity assessment of undiluted and diluted hydrogel extracts in MG-63 human osteoblast **(A,B)** and L929 mouse fibroblast **(C,D)** cell cultures. **(A)** 2 h extracts for MG-63; **(B)** 24 h extracts for MG-63; **(C)** 2 h extracts for L929; **(D)** 24 h extracts for L929. The black dotted line corresponds to the control (100%, cells cultivated in standard cultivation media), and the red dotted line represents cytotoxicity control DMSO (viability 29.57 ± 6.54%), *n* = 6.

However, results revealed that already all 2 h extracts caused some cytotoxic effects on L929 and MG-63 cell lines, with the exception of HA_H_SrRan extract (Figures 11A, C).

Reduction of cell viability was observed for both undiluted and 1:10 diluted extracts. When 1:100 extract was used, the impact on cell viability was less pronounced as the cell viability was reduced by less than 25% in both cell lines. In the case of 24 h extracts, strong cytotoxic activity was observed when undiluted extracts were added to the cell cultures (except HA_H_SrRan). Extracts in both cell lines reduced cell viability by more than 60%. In the case of 24 h extracts, the negative effect on cell viability was less pronounced, compared to 2 h extracts (Figures 11B, D). Overall, the hydrogel extract test clearly demonstrated that leaking substances negatively affected the cell viability in addition to the effect that was seen already after 2 h extraction. The cytotoxic effect of 24 h extracts indicated that the leakage of potentially toxic components of the biomaterial continues.

**FIGURE 12:**
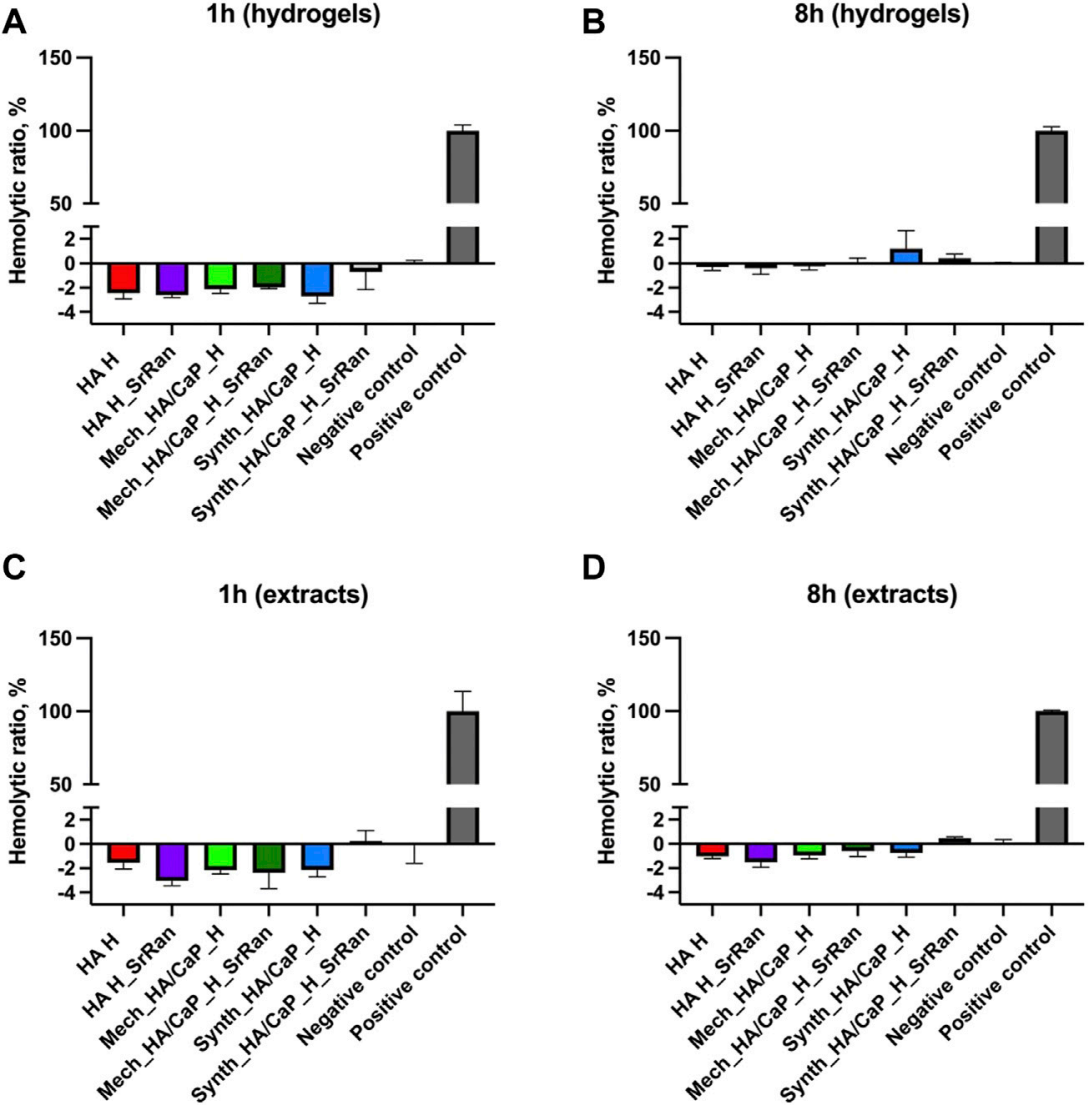
Hemocompatibility of hydrogels **(A,B)** and hydrogel extracts **(C,D)**; positive control: deionized water; negative control: PBS (pH 7.4); *n* = 3.

Obtained *in vitro* results showed that the effects on cell viability varied between the cell lines. A negative impact on cell viability was observed for hydrogel extracts, with cytotoxicity decreasing if extracts were diluted. Hence, neither hydrogels nor their extracts induced hemolysis even during the prolonged incubation periods (Figure 12).

Results indicate that hydrogels or compounds leaking from them do not damage cell membranes. The toxicity in actively dividing cells as fibroblasts and osteoblasts might be due to the compounds leaking from the hydrogels that are capable to interfere with cellular metabolism or due to the alkaline pH of the hydrogels (Section 3.2).

## 4 Conclusion

BDDE cross-linked hyaluronic acid/calcium phosphate (HA/CaP) composites were successfully developed and modified with antiosteoporotic drug-strontium ranelate (SrRan). During the study, it was found that the properties of the materials strongly depend on the CaP presence in the hydrogels as well as on the approach of CaP particle incorporation into the HA matrix. FT-IR results did not confirm any new chemical bonds or molecular interactions caused by SrRan or Ca^2+^ presence in the hydrogels. Pore size distribution in the composites, pore wall thickness, as well as pore interconnectivity, were analyzed using µCT, and the obtained results revealed that the developed HA-containing hydrogels could ensure the migration of bone cells and the formation of new bone tissue, as the pore size was in the range of 100–400 μm.

Obtained results revealed that the addition of CaP, as well as the route of nanoparticle introduction into the HA matrix, highly influenced the gel fraction of composites. Furthermore, gel fraction strongly affected the pH changes and swelling of the hydrogels during their neutralization process, as well as the swelling behavior of the lyophilized samples. It was also found that although the route of CaP nanoparticle incorporation into the HA matrix had a significant effect on the hydrogel gel fraction and swelling behavior, it had a negligible effect on the release kinetics of the SrRan and Sr^2+^. The addition of CaP to the hydrogels retarded the SrRan release by ∼60% if compared with pure HA hydrogels.

Even though 3T3 cell viability in all experiments was higher than 70%, the highest cell viability (>80%) was found for HA hydrogels with and without SrRan. Moreover, positive effects of SrRan on 3T3 cells were demonstrated, showing a significant increase (up to 50%) in cell viability if the used SrRan concentrations ranged from 0.05–0.2 μg/ml.

Finally, obtained *in vitro* biocompatibility results revealed that the effects on cell viability significantly varied between the cell lines (3T3, L929, and MG-63), emphasizing the importance of cell line choice for the biomaterial cytotoxicity assessment.

## Data Availability

The raw data supporting the conclusion of this article will be made available by the authors, without undue reservation.

## References

[B1] Abbasi N. Hamlet S. Love R. M. Nguyen N.-T. (2020). Porous Scaffolds for Bone Regeneration. J. Sci. Adv. Mater. Devices 5, 1–9. 10.1016/j.jsamd.2020.01.007

[B2] Aderibigbe B. Aderibigbe I. Popoola P. (2017). Design and Biological Evaluation of Delivery Systems Containing Bisphosphonates. Pharmaceutics 9, 2–24. 10.3390/pharmaceutics9010002 PMC5374368 28035945

[B3] Ahmadian E. Eftekhari A. Dizaj S. M. Sharifi S. Mokhtarpour M. Nasibova A. N. (2019). The Effect of Hyaluronic Acid Hydrogels on Dental Pulp Stem Cells Behavior. Int. J. Biol. Macromol. 140, 245–254. 10.1016/j.ijbiomac.2019.08.119 31419560

[B4] Alkrad J. A. Mrestani Y. Stroehl D. Wartewig S. Neubert R. (2003). Characterization of Enzymatically Digested Hyaluronic Acid Using NMR, Raman, IR, and UV-VIS Spectroscopies. J. Pharm. Biomed. Analysis 31, 545–550. 10.1016/S0731-7085(02)00682-9 12615242

[B5] Andrade Neto D. M. Carvalho E. V. Rodrigues E. A. Feitosa V. P. Sauro S. Mele G. (2016). Novel Hydroxyapatite Nanorods Improve Anti-caries Efficacy of Enamel Infiltrants. Dent. Mater. 32, 784–793. 10.1016/j.dental.2016.03.026 27068739

[B6] Ansari M. (2019). Bone Tissue Regeneration: Biology, Strategies and Interface Studies. Prog. Biomater. 8, 223–237. 10.1007/s40204-019-00125-z 31768895 PMC6930319

[B7] Asafo-Adjei T. A. Chen A. J. Najarzadeh A. Puleo D. A. (2016). Advances in Controlled Drug Delivery for Treatment of Osteoporosis. Curr. Osteoporos. Rep. 14, 226–238. 10.1007/s11914-016-0321-4 27502334 PMC5035217

[B8] Briot K. Roux C. (2005). Strontium Ranelate: State of the Art. Women's Health 1, 15–21. 10.1517/17455057.1.1.01510.2217/17455057.1.1.15 19803941

[B9] Caverzasio J. Thouverey C. (2011). Activation of FGF Receptors Is a New Mechanism by Which Strontium Ranelate Induces Osteoblastic Cell Growth. Cell. Physiol. biochem. 27, 243–250. 10.1159/000327950 21471713

[B10] Chang S.-H. Custer P. L. Mohadjer Y. Scott E. (2009). Use of Lorenz Titanium Implants in Orbital Fracture Repair. Ophthal. Plast. Reconstr. Surg. 25, 119–122. 10.1097/IOP.0b013e31819ac7c5 19300154

[B11] Chanthick C. Thongboonkerd V. (2020). Effects of Hyaluronic Acid on Calcium Oxalate Crystallization, Growth, Aggregation, Adhesion on Renal Tubular Cells, and Invasion through Extracellular Matrix. Curr. Dev. Nutr. 4, 13. 10.1093/cdn/nzaa040_013 30701361

[B12] Chen S. Zhao R. Xing Z. Shang T. Yang X. Zhu X. (2021). Strontium Combined with Bioceramics for Osteoporotic Bone Repair: Oral Intake or as a Dopant? Appl. Mater. Today 22, 100927. 10.1016/j.apmt.2020.100927

[B13] Chen Z.-H. Ren X.-L. Zhou H.-H. Li X.-D. (2012). The Role of Hyaluronic Acid in Biomineralization. Front. Mat. Sci. 6, 283–296. 10.1007/s11706-012-0182-4

[B14] Chiang C.-W. Hsiao Y.-C. Jheng P.-R. Chen C.-H. Manga Y. B. Lekha R. (2021). Strontium Ranelate-Laden Near-Infrared Photothermal-Inspired Methylcellulose Hydrogel for Arthritis Treatment. Mater. Sci. Eng. C 123, 111980. 10.1016/j.msec.2021.111980 33812608

[B15] Chindamo G. Sapino S. Peira E. Chirio D. Gonzalez M. C. Gallarate M. (2020). Bone Diseases: Current Approach and Future Perspectives in Drug Delivery Systems for Bone Targeted Therapeutics. Nanomaterials 10, 875. 10.3390/nano10050875 PMC7279399 32370009

[B16] Choi S. Lee J. S. Shin J. Lee M. S. Kang D. Hwang N. S. (2020). Osteoconductive Hybrid Hyaluronic Acid Hydrogel Patch for Effective Bone Formation. J. Control. Release 327, 571–583. 10.1016/j.jconrel.2020.09.006 32905801

[B17] Dreiss C. A. (2020). Hydrogel Design Strategies for Drug Delivery. Curr. Opin. Colloid & Interface Sci. 48, 1–17. 10.1016/j.cocis.2020.02.001

[B18] Ellerbrock R. H. Gerke H. H. (2021). FTIR Spectral Band Shifts Explained by OM-Cation Interactions. J. Plant Nutr. Soil Sci. 184, 388–397. 10.1002/jpln.202100056

[B19] Er K. Polat Z. A. Özan F. Taşdemir T. Sezer U. Siso Ş. H. (2008). Cytotoxicity Analysis of Strontium Ranelate on Cultured Human Periodontal Ligament Fibroblasts: A Preliminary Report. J. Formos. Med. Assoc. 107, 609–615. 10.1016/S0929-6646(08)60178-3 18678544

[B20] Fang Y. Shi L. Duan Z. Rohani S. (2021). Hyaluronic Acid Hydrogels, as a Biological Macromolecule-Based Platform for Stem Cells Delivery and Their Fate Control: A Review. Int. J. Biol. Macromol. 189, 554–566. 10.1016/j.ijbiomac.2021.08.140 34437920

[B21] Farbod K. Nejadnik M. R. Jansen J. A. Leeuwenburgh S. C. G. (2014). Interactions between Inorganic and Organic Phases in Bone Tissue as a Source of Inspiration for Design of Novel Nanocomposites. Tissue Eng. Part B Rev. 20, 173–188. 10.1089/ten.teb.2013.0221 23902258

[B22] Farrell K. B. Karpeisky A. Thamm D. H. Zinnen S. (2018). Bisphosphonate Conjugation for Bone Specific Drug Targeting. Bone Rep. 9, 47–60. 10.1016/j.bonr.2018.06.007 29992180 PMC6037665

[B23] Fernandes G. Vanyo S. T. Alsharif S. B. A. Andreana S. Visser M. B. Dziak R. (2019). Strontium Effects on Human Gingival Fibroblasts. J. Oral Implantol. 45, 274–280. 10.1563/aaid-joi-D-18-00253 31216254

[B24] Grenier J. Duval H. Barou F. Lv P. David B. Letourneur D. (2019). Mechanisms of Pore Formation in Hydrogel Scaffolds Textured by Freeze-Drying. Acta Biomater. 94, 195–203. 10.1016/j.actbio.2019.05.070 31154055

[B25] Gu S. Cheng G. Yang T. Ren X. Gao G. (2017). Mechanical and Rheological Behavior of Hybrid Cross-Linked Polyacrylamide/cationic Micelle Hydrogels. Macromol. Mat. Eng. 302 (12), 1700402. 10.1002/mame.201700402

[B26] Hamad G. M. H. Taha T. E. Hafez E. El Sohaimy S. (2017). Physicochemical, Molecular and Functional Characteristics of Hyaluronic Acid as a Functional Food. Am. J. Food Technol. 12, 72–85. 10.3923/ajft.2017.72.85

[B27] Hildebrand T. Rüegsegger P. (1997). A New Method for the Model-independent Assessment of Thickness in Three-Dimensional Images. J. Microsc. 185, 67–75. 10.1046/j.1365-2818.1997.1340694.x

[B28] Hoare T. R. Kohane D. S. (2008). Hydrogels in Drug Delivery: Progress and Challenges. Polymer 49, 1993–2007. 10.1016/j.polymer.2008.01.027

[B29] Huang X. Brazel C. S. (2001). On the Importance and Mechanisms of Burst Release in Matrix-Controlled Drug Delivery Systems. J. Control. Release 73, 121–136. 10.1016/S0168-3659(01)00248-6 11516493

[B30] Karageorgiou V. Kaplan D. (2005). Porosity of 3D Biomaterial Scaffolds and Osteogenesis. Biomaterials 26, 5474–5491. 10.1016/j.biomaterials.2005.02.002 15860204

[B31] Kenne L. Gohil S. Nilsson E. M. Karlsson A. Ericsson D. Helander Kenne A. (2013). Modification and Cross-Linking Parameters in Hyaluronic Acid Hydrogels-Definitions and Analytical Methods. Carbohydr. Polym. 91, 410–418. 10.1016/j.carbpol.2012.08.066 23044151

[B32] Kim J.-M. Lin C. Stavre Z. Greenblatt M. B. Shim J.-H. (2020). Osteoblast-osteoclast Communication and Bone Homeostasis. Cells 9, 2073. 10.3390/cells9092073 PMC7564526 32927921

[B33] Kim J.-T. Lee D.-Y. Kim Y.-H. Lee I.-K. Song Y.-S. (2012). Effect of pH on Swelling Property of Hyaluronic Acid Hydrogels for Smart Drug Delivery Systems. J. Sens. Sci. Technol. 21, 256–262. 10.5369/JSST.2012.21.4.256

[B34] Kruse C. R. Singh M. Targosinski S. Sinha I. Sørensen J. A. Eriksson E. (2017). The Effect of pH on Cell Viability, Cell Migration, Cell Proliferation, Wound Closure, and Wound Reepithelialization: *In Vitro* and *In Vivo* Study. Wound Rep Reg 25, 260–269. 10.1111/wrr.12526 28370923

[B35] Kyllönen L. D’Este M. Alini M. Eglin D. (2015). Local Drug Delivery for Enhancing Fracture Healing in Osteoporotic Bone. Acta Biomater. 11, 412–434. 10.1016/j.actbio.2014.09.006 25218339

[B36] Lapčík L. Lapčík L. De Smedt S. Demeester J. Chabreček P. (1998). Hyaluronan: Preparation, Structure, Properties, and Applications. Chem. Rev. 98, 2663–2684. 10.1021/cr941199z 11848975

[B37] Li J. Mooney D. J. (2016). Designing Hydrogels for Controlled Drug Delivery. Nat. Rev. Mat. 1. 10.1038/natrevmats.2016.71 PMC5898614 29657852

[B38] Lin X. Patil S. Gao Y.-G. Qian A. (2020). The Bone Extracellular Matrix in Bone Formation and Regeneration. Front. Pharmacol. 11, 757. 10.3389/fphar.2020.00757 32528290 PMC7264100

[B39] Loca D. Smirnova A. Locs J. Dubnika A. Vecstaudza J. Stipniece L. (2018). Development of Local Strontium Ranelate Delivery Systems and Long Term *In Vitro* Drug Release Studies in Osteogenic Medium. Sci. Rep. 8, 1–10. 10.1038/s41598-018-35197-7 30425295 PMC6233163

[B40] Lowry K. M. Beavers E. M. (1994). Thermal Stability of Sodium Hyaluronate in Aqueous Solution. J. Biomed. Mat. Res. 28, 1239–1244. 10.1002/jbm.820281014 7829553

[B41] Mandal K. Raz-Ben Aroush D. Graber Z. T. Wu B. Park C. Y. Fredberg J. J. (2019). Soft Hyaluronic Gels Promote Cell Spreading, Stress Fibers, Focal Adhesion, and Membrane Tension by Phosphoinositide Signaling, Not Traction Force. ACS Nano 13, 203–214. 10.1021/acsnano.8b05286 30500159 PMC6511072

[B42] Mao Z. Li Y. Yang Y. Fang Z. Chen X. Wang Y. (2018). Osteoinductivity and Antibacterial Properties of Strontium Ranelate-Loaded Poly(Lactic-Co-Glycolic Acid) Microspheres with Assembled Silver and Hydroxyapatite Nanoparticles. Front. Pharmacol. 9. 10.3389/fphar.2018.00368 PMC5915458 29720940

[B43] Mariggiò M. A. Cassano A. Vinella A. Vincenti A. Fumarulo R. Muzio L. L. (2009). Enhancement of Fibroblast Proliferation, Collagen Biosynthesis and Production of Growth Factors as a Result of Combining Sodium Hyaluronate and Aminoacids. Int. J. Immunopathol. Pharmacol. 22, 485–492. 10.1177/039463200902200225 19505400

[B44] Marx D. Rahimnejad Yazdi A. Papini M. Towler M. (2020). A Review of the Latest Insights into the Mechanism of Action of Strontium in Bone. Bone Rep. 12, 100273. 10.1016/j.bonr.2020.100273 32395571 PMC7210412

[B45] Mcneil S. E. Hobson S. A. Nipper V. Rodland K. D. (1998). Functional Calcium-Sensing Receptors in Rat Fibroblasts Are Required for Activation of SRC Kinase and Mitogen-Activated Protein Kinase in Response to Extracellular Calcium. J. Biol. Chem. 273, 1114–1120. 10.1074/jbc.273.2.1114 9422777

[B46] Nair B. P. Sindhu M. Nair P. D. (2016). Polycaprolactone-laponite Composite Scaffold Releasing Strontium Ranelate for Bone Tissue Engineering Applications. Colloids Surfaces B Biointerfaces 143, 423–430. 10.1016/j.colsurfb.2016.03.033 27037779

[B47] Nardone V. Fabbri S. Marini F. Zonefrati R. Galli G. Carossino A. (2012). Osteodifferentiation of Human Preadipocytes Induced by Strontium Released from Hydrogels. Int. J. Biomaterials 2012, 1–10. 10.1155/2012/865291 PMC3423935 22927856

[B48] Newman M. R. Benoit D. S. (2016). Local and Targeted Drug Delivery for Bone Regeneration. Curr. Opin. Biotechnol. 40, 125–132. 10.1016/j.copbio.2016.02.029 27064433 PMC4975663

[B49] Papathanasiou K. E. Turhanen P. Brückner S. I. Brunner E. Demadis K. D. (2017). Smart, Programmable and Responsive Injectable Hydrogels for Controlled Release of Cargo Osteoporosis Drugs. Sci. Rep. 7, 2–10. 10.1038/s41598-017-04956-3 28684783 PMC5500573

[B50] Pemmer B. Hofstaetter J. G. Meirer F. Smolek S. Wobrauschek P. Simon R. (2011). Increased Strontium Uptake in Trabecular Bone of Ovariectomized Calcium-Deficient Rats Treated with Strontium Ranelate or Strontium Chloride. J. Synchrotron Radiat. 18, 835–841. 10.1107/S090904951103038X 21997907

[B51] Peng S. Lai Z.-T. Hong D.-W. Chu I.-M. Lai P.-L. (2017). Controlled Release of Strontium through Neutralization Reaction within a Methoxy(Polyethylene Glycol)-Polyesterc Hydrogel. J. Appl. Biomaterials Funct. Mater. 15, 162–169. 10.5301/jabfm.5000313 27716871

[B52] Pilmane M. Salma-Ancane K. Loca D. Locs J. Berzina-Cimdina L. (2017). Strontium and Strontium Ranelate: Historical Review of Some of Their Functions. Mater. Sci. Eng. C 78, 1222–1230. 10.1016/j.msec.2017.05.042 28575961

[B53] Place E. S. Rojo L. Gentleman E. Sardinha J. P. Stevens M. M. (2011). Strontium- and Zinc-Alginate Hydrogels for Bone Tissue Engineering. Tissue Eng. Part A 17, 2713–2722. 10.1089/ten.tea.2011.0059 21682547

[B54] Sadegh A. B. (2018). Strontium Ranelate Using for the Treatment of Postmenopausal Osteoporosis. Biomed. J. Sci. Tech. Res. 5, 001–007. 10.26717/bjstr.2018.05.001228

[B55] Salma K. Berzina-Cimdina L. Borodajenko N. (2010). Calcium Phosphate Bioceramics Prepared from Wet Chemically Precipitated Powders. Pac 4, 45–51. 10.2298/PAC1001045S

[B56] Salma-Ancane K. Sceglovs A. Tracuma E. Wychowaniec J. K. Aunina K. Ramata-Stunda A. (2022). Effect of Crosslinking Strategy on the Biological, Antibacterial and Physicochemical Performance of Hyaluronic Acid and Ɛ-Polylysine Based Hydrogels. Int. J. Biol. Macromol. 208, 995–1008. 10.1016/j.ijbiomac.2022.03.207 35378161

[B57] Sokolova M. Locs J. Loca D. (2017). Hyaluronan Hydrogel/calcium Phosphates Composites for Medical Application. Key Eng. Mat. 721, 219–223. 10.4028/www.scientific.net/KEM.721.219

[B58] Stapleton M. Sawamoto K. Alméciga-Díaz C. Mackenzie W. Mason R. Orii T. (2017). Development of Bone Targeting Drugs. Ijms 18, 1345. 10.3390/ijms18071345 PMC5535838 28644392

[B59] Stojkov G. Niyazov Z. Picchioni F. Bose R. K. (2021). Relationship between Structure and Rheology of Hydrogels for Various Applications. Gels 7 (4), 255. 10.3390/gels7040255 34940315 PMC8700820

[B60] Stolarczyk M. Matera-Witkiewicz A. Wolska A. Krupińska M. Mikołajczyk A. Pyra A. (2021). Synthesis, Crystal Structure, and Biological Evaluation of Novel 5-Hydroxymethylpyrimidines. Materials 14, 6916. 10.3390/ma14226916 34832318 PMC8618934

[B61] Wei Y. Chang Y.-H. Liu C.-J. Chung R.-J. (2018). Integrated Oxidized-Hyaluronic Acid/Collagen Hydrogel with β-TCP Using Proanthocyanidins as a Crosslinker for Drug Delivery. Pharmaceutics 10, 37. 10.3390/pharmaceutics10020037 PMC6030783 29561754

[B62] Wychowaniec J. K. Litowczenko J. Tadyszak K. Natu V. Aparicio C. Peplińska B. (2020). Unique Cellular Network Formation Guided by Heterostructures Based on Reduced Graphene Oxide - Ti3C2Tx MXene Hydrogels. Acta Biomater. 115, 104–115. 10.1016/j.actbio.2020.08.010 32795646

[B63] Xie Y. Zhang L. Xiong Q. Gao Y. Ge W. Tang P. (2019). Bench-to-bedside Strategies for Osteoporotic Fracture: From Osteoimmunology to Mechanosensation. Bone Res. 7. 10.1038/s41413-019-0066-7 PMC6804735 31646015

[B64] Yamaguchi M. Neale Weitzmann M. (2012). The Intact Strontium Ranelate Complex Stimulates Osteoblastogenesis and Suppresses Osteoclastogenesis by Antagonizing NF-κB Activation. Mol. Cell. Biochem. 359, 399–407. 10.1007/s11010-011-1034-8 21874315

[B65] Yang B. Guo X. Zang H. Liu J. (2015). Determination of Modification Degree in BDDE-Modified Hyaluronic Acid Hydrogel by SEC/MS. Carbohydr. Polym. 131, 233–239. 10.1016/j.carbpol.2015.05.050 26256180

[B66] Yin X. Zhou C. Li J. Liu R. Shi B. Yuan Q. (2019). Autophagy in Bone Homeostasis and the Onset of Osteoporosis. Bone Res. 7. 10.1038/s41413-019-0058-7 PMC6804951 31666998

[B67] Žádníková P. Šínová R. Pavlík V. Šimek M. Šafránková B. Hermannová M. (2022). The Degradation of Hyaluronan in the Skin. Biomol 12, 251. 10.3390/biom12020251 PMC8961566 35204753

[B68] Zhai P. Peng X. Li B. Liu Y. Sun H. Li X. (2020). The Application of Hyaluronic Acid in Bone Regeneration. Int. J. Biol. Macromol. 151, 1224–1239. 10.1016/j.ijbiomac.2019.10.169 31751713

[B69] Zhang J. N. Chen B. Z. Ashfaq M. Zhang X. P. Guo X. D. (2018). Development of a BDDE-Crosslinked Hyaluronic Acid Based Microneedles Patch as a Dermal Filler for Anti-ageing Treatment. J. Industrial Eng. Chem. 65, 363–369. 10.1016/j.jiec.2018.05.007

[B70] Zhao D. Wang X. Tie C. Cheng B. Yang S. Sun Z. (2021). Bio-functional Strontium-Containing Photocrosslinked Alginate Hydrogels for Promoting the Osteogenic Behaviors. Mater. Sci. Eng. C 126, 112130. 10.1016/j.msec.2021.112130 34082947

[B71] Zhen W. Jiang C. Feng B. Xiaojiang S. Jianxi L. Li L. (2010). Role of the Porous Structure of the Bioceramic Scaffolds in Bone Tissue Engineering. Nat. Prec. 10.1038/npre.2010.4148.1

