# Supplementary material for: Calcium Phosphate/Hyaluronic Acid Composite Hydrogels for Local Antiosteoporotic Drug Delivery
Source: Front Bioeng Biotechnol. 2022 Jul 5;10:917765. doi: 10.3389/fbioe.2022.917765 (PMC9294454; doi:10.3389/fbioe.2022.917765)
Supplement: Supplementary file 1 [file DataSheet1.docx]

Supplementary Material

**
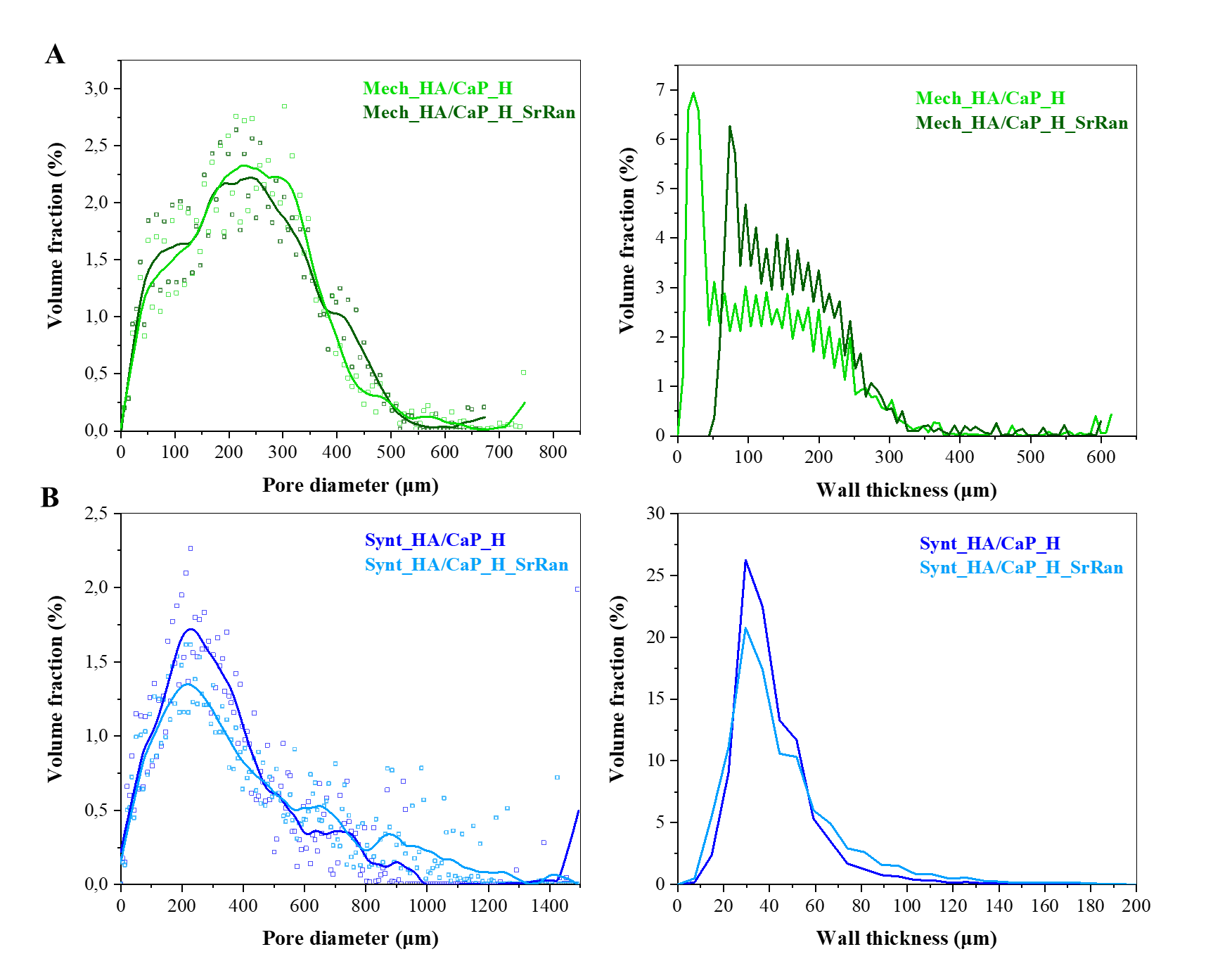
**

**Fig. S1** Characterization of microstructure of CaP containing hydrogels: (A) Volume fraction of pore size in the lyophilized hydrogel; (B) Volume fraction of pore wall thickness in the lyophilized hydrogel

**
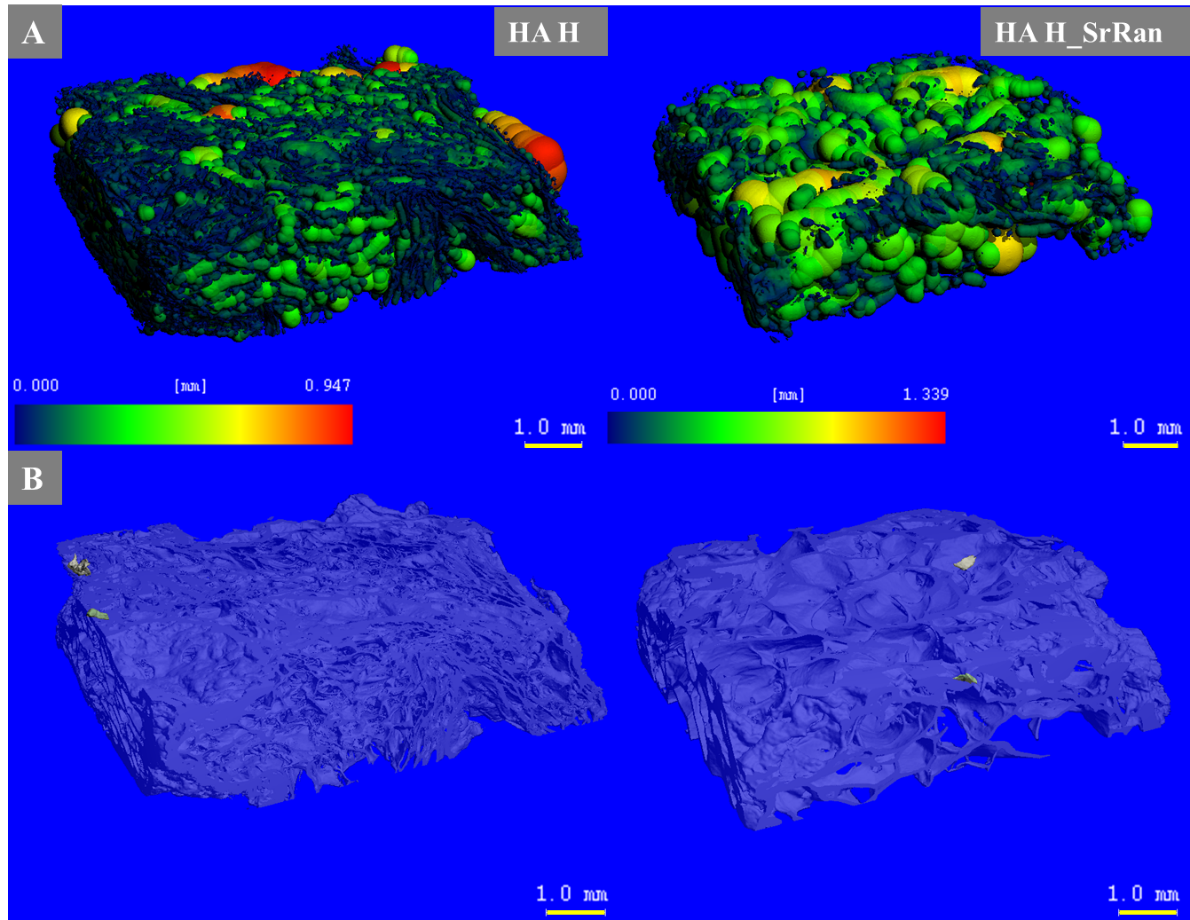
**

**
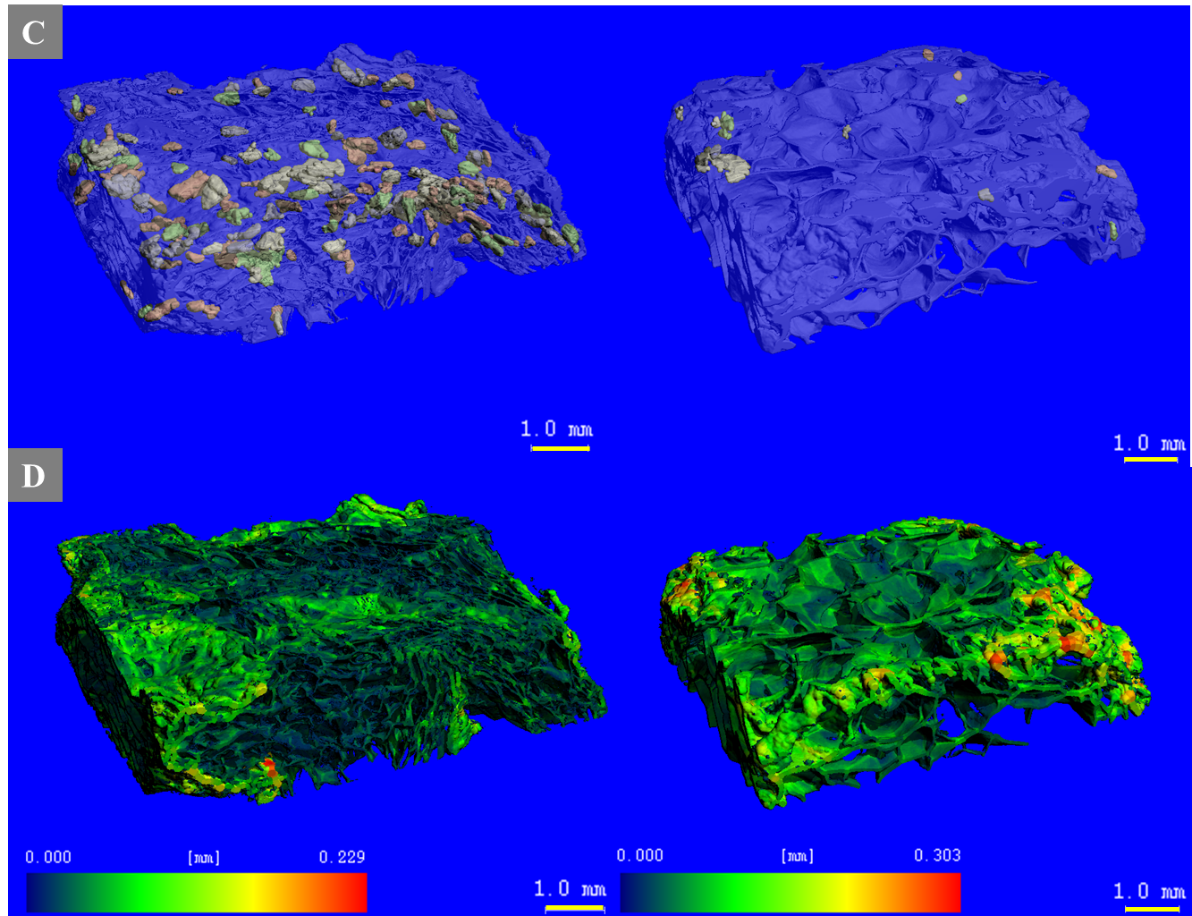
**

**Fig. S2** Microstructure of HA hydrogels: (A) Heatmap of pore size distribution; (B) Closed pores; (C) Slightly connected pores; (D) Heatmap of pore wall thickness

**
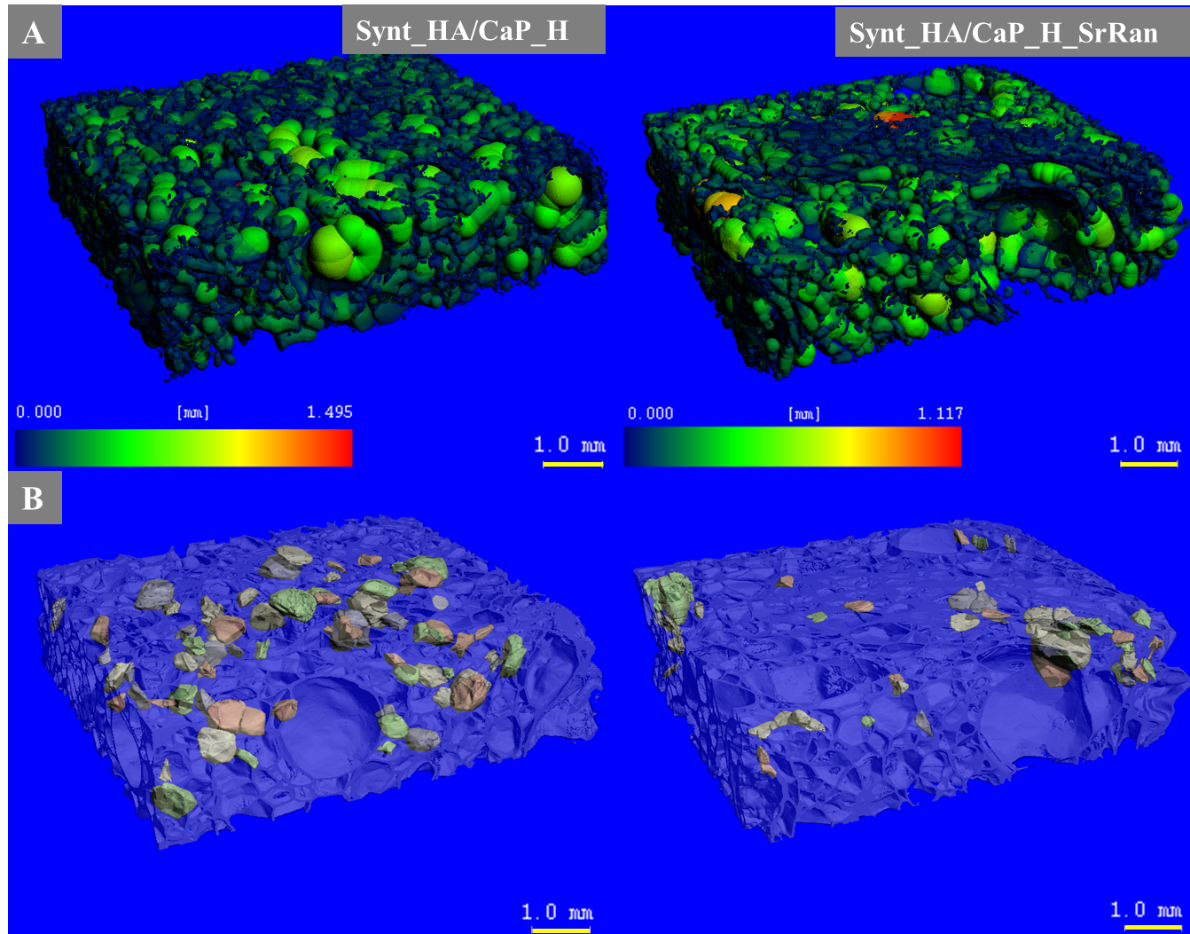

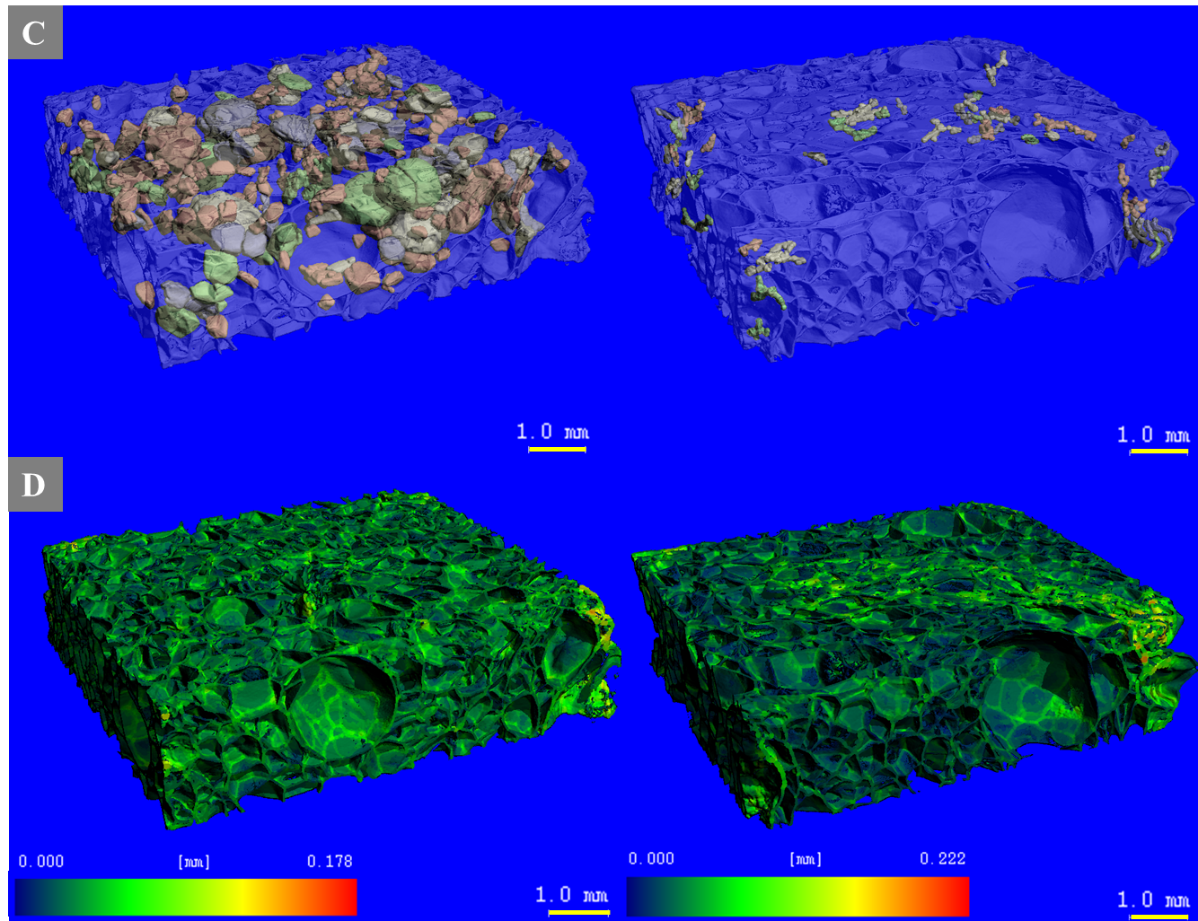
**

**Fig. S3** Microstructure of Synt_HA/CaP and Synt-HA/CaP_SrRan hydrogels: (A) Heatmap of pore size distribution; (B) Closed pores; (C) Slightly connected pores; (D) Heatmap of pore wall thickness


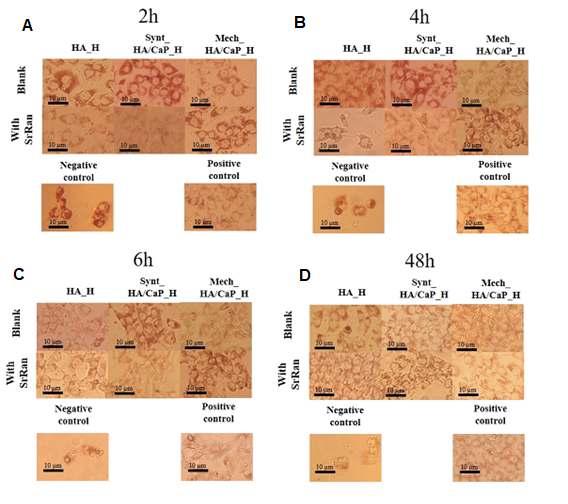


**Fig. S4** Morphology of cells after incubation with prepared hydrogel extracts: (A) 2h, (B) 4h, (C) 6h, (D) 48h.

**
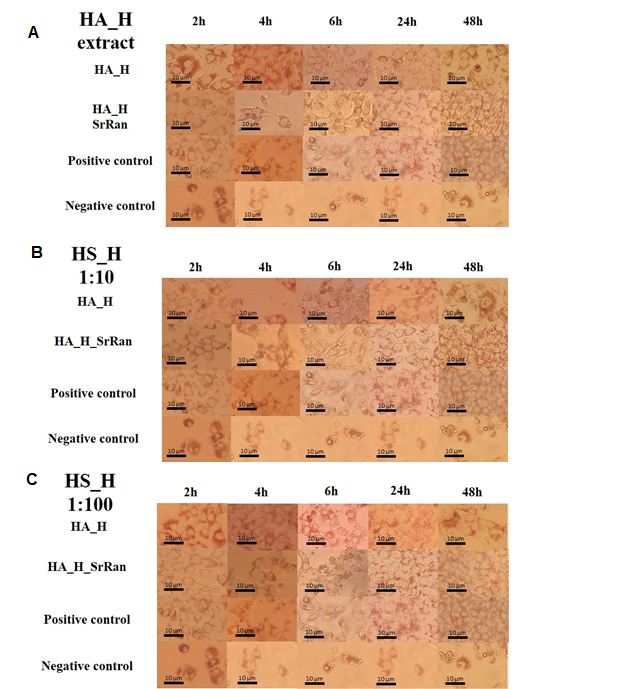
**

**Fig. S5.** Cell morphology at different time points for HA hydrogels: (A) Extracts; (B) Extract dilution 1:10; (C) Extract dilution 1:100


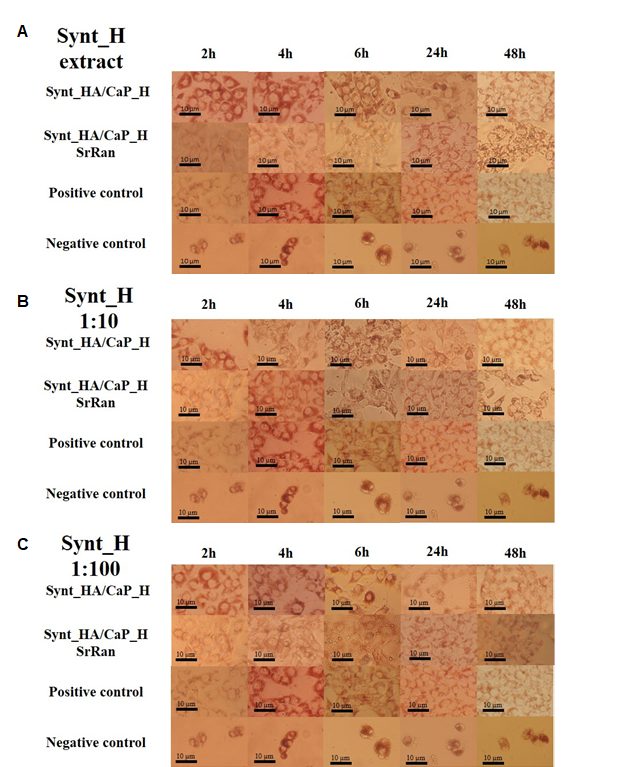


**Fig. S6.** Cell morphology at different time points for Synt_HA/CaP composite hydrogels: (A) Extracts; (B) Extract dilution 1:10; (C) Extract dilution 1:100

**
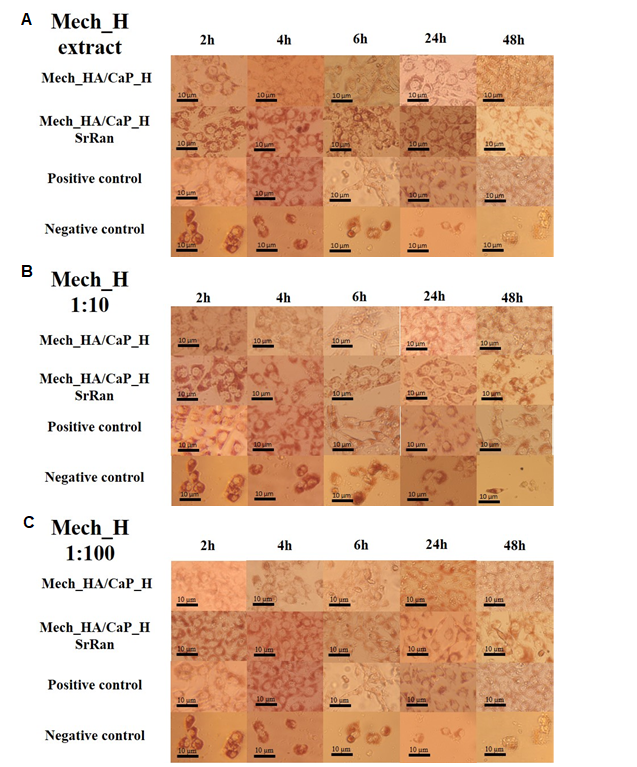
**

**Fig. S7.** Cell morphology at different time points for Mech_HA/CaP composite hydrogels; (A) Extracts; (B) Extract dilution 1:10; (C) Extract dilution 1:100
